# Organelle genomes reveal adaptive evolution and phylogenetic position of the endangered *Primula mallophylla*

**DOI:** 10.3389/fpls.2025.1653128

**Published:** 2025-10-28

**Authors:** Wenqiao Li, Le Wang, Youwei Zuo, Huan Zhang, Xiao Zhang, Lingxiang Yang, Jiabin Zhang, Fengyuan Zheng, Hongping Deng

**Affiliations:** ^1^ School of Life Sciences, Southwest University, Chongqing, China; ^2^ Administration Center of Chongqing Dabashan National Nature Reserve, Chongqing, China

**Keywords:** *Primula mallophylla*, mitochondrial genome, chloroplast genome, RNA editing event, adaptive evolution, phylogeny

## Abstract

**Introduction:**

Species of *Primula* section *Proliferae* are predominantly distributed in alpine environments above 2,000 meters and represent an important group for studying environmental adaptation and phylogenetic evolution. *Primula mallophylla*, holds important ecological, ornamental, and conservation value. However, genomic resources for this species remain unavailable, hindering further research on its genetic evolution and conservation strategies.

**Methods:**

In this study, we sequenced and assembled the complete mitochondrial and chloroplast genomes of *P. mallophylla* for the first time. Comprehensive analyses were conducted on their structural characteristics, repetitive sequences, RNA editing site prediction, codon usage bias, intracellular gene transfer, phylogenetic inference, and selective pressure.

**Results:**

The results show that both the mitochondrial and chloroplast genomes exhibit typical master circular structures, with sizes of 340,219 bp and 150,733 bp, respectively. The mitochondrial genome has more abundant repetitive sequences and has undergone genomic rearrangements. There are only 9 MTPTs between mitochondria and chloroplasts, totaling 2,028 bp. In the regulation of mitochondrial genome expression, we predicted 475 RNA editing sites, with *ccmB* and *mttB* showing the highest potential editing frequencies. We found that *P. mallophylla* exhibits similar codon usage bias to most plants, and based on ENC-GC3s analysis, some genes appear to be under natural selection. Chloroplast genes *rpl2*, *rpl22*, *rbcL*, and *ndhB* exhibit branch-specific positive selection in Primula, reflecting adaptive evolution of photosynthesis and protein synthesis functions in high-altitude environments. Based on phylogenetic trees constructed from PCGs, Primulaceae are most closely related to Ebenaceae. Furthermore, phylogenetic analysis based on chloroplast genomes and PCGs of *Primula* species showed that sect. *Proliferae* is not monophyletic, with *P. stenodonta* being a sister species to *P. mallophylla*.

**Discussion:**

These findings provide crucial genomic resources and insights into the adaptive evolution of *P. mallophylla*, while also clarifying phylogenetic relationships within sect. *Proliferae* and Primulaceae, thereby offering valuable guidance for conservation strategies and further evolutionary studies.

## 1 Introduction

*Primula* is the largest genus in the Primulaceae, comprising over 500 species primarily distributed in alpine regions and temperate zones of the Northern Hemisphere. In China, more than 300 species are found, most of which are endemic, with the Eastern Himalaya-Hengduan Mountains as the hotspot area (Richards, 2003; Hu and Kelso, 1996; Hu, 1998). This genus is of great ornamental value in horticulture and floriculture due to its long flowering period, colorful inflorescences, and distinctive shape, and is regarded as one of the greatest genera of garden plants in the world (Richards, 2002; Yan et al., 2010). In addition, the species of *Primula* have a long tradition of folk medicinal use, exhibiting significant therapeutic effects in treating various conditions, including rheumatic pain, headaches, insomnia, nervous tension, and ocular diseases (Budzianowski et al., 2005; Başbülbül et al., 2008; Majid et al., 2014; Khan et al., 2022).

Species of *Primula* are cross-pollinated plants and serve as an ideal model for evolution (Darwin, 1877; Mast et al., 2006; Gilmartin, 2015). Its systematic position and the phylogenetic relationships within the genus have long been focal points. Schott (1851) divided *Primula* into two subgenera for the first time, but this classification was only adopted by a few scholars. Pax (1889) divided *Primula* into 21 sections, but the boundaries between sections were not clear. Subsequently, Smith and Fletcher (1947) systematically classified the *Primula* into 31 sections. To better express the genetic relationship between sections, Wendelbo (1961) elevated some sections classified by Smith and Fletcher to the level of subgenus, and classified its 31 sections into 7 subgenera. Halda (1992) divided *Primula* into 8 subgenera, and took *Omphalogramma* as a subgenus within *Primula*. Then, Richards (2003) revised the *Primula* into a six-subgenera system, and divided it into 38 sections. However, Hu (1990, 1994, 1998) believed that there were cross and overlap among the sections of *Primula*, and their differentiation had not yet reached the subgenus level. Therefore, they divided the genus into 30 sections, including 24 sections in China. The above research has yielded substantial insights into the phylogenetic development of *Primula* taxa. Nevertheless, the interspecific hybridization, parallel evolution, gene introgression and other complex phenomena occur frequently in the long-term evolution of these species (Xie et al., 2017; Stubbs et al., 2024), which makes it difficult to clarify the phylogenetic relationship of the *Primula*, and the results of molecular phylogeny within the genus are in great conflict with the traditional classification system (Mast et al., 2001, Mast et al., 2006; Trift et al., 2002; Schmidt-Lebuhn et al., 2012). Numerous studies based on phylogenetic analyses of chloroplast genomes have demonstrated that some sections (such as: sect. *Proliferae*, sect. *Crystallophlomis*, sect. *Monocarpicae*, sect. *Obconicolisteri*) were not a monophyletic group (Li, 2022; Xie et al., 2023). Thus, additional genomic data are required to clarify the evolutionary history of the *Primula* further.

The organelle genomes of plants, including the mitochondrial genome (mtDNA) and chloroplast genome (cpDNA), are indispensable for the investigation of plant evolution, physiological regulation, and phylogeny (Maréchal and Brisson, 2010). In contrast to the nuclear genome, organelle genes have a low mutation rate and are predominantly maternally inherited, making them widely used in species identification, phylogenetic analysis, and studies of adaptive evolution (Wang et al., 2024a). The chloroplast genome exhibits a conserved structure, typically comprising a quadripartite structure with a large single-copy region (LSC), a small single-copy region (SSC), and two inverted repeat regions (IRs), and contains numerous functional genes that are involved in photosynthesis and key protein synthesis (Liu et al., 2024a). In contrast, the mitochondrial genome exhibits greater variability in both structure and size, including linear, circular, more complex branching or reticular structures, and is mainly involved in the respiratory chain, protein synthesis, and energy metabolism-related functions (Xia et al., 2023; Liu et al., 2024b; Yong et al., 2025). However, the abundance of numerous repeat sequences, insertion fragments, and exogenous DNA integration (including fragments from nuclear and chloroplast genomes) among its genes, especially post-transcriptional RNA editing events, may regulate gene expression and thus affect adaptation to environmental stresses. Thus, the mitochondrial genome has a unique role to play in the evolution of plant adaptations and the differentiation of species (Li et al., 2024c, Li et al., 2025). Nonetheless, systematic studies of organelle genomes in the genus *Primula* remain limited, with fewer than five mitochondrial genomes and fewer than 100 chloroplast genomes reported, which has constrained a deeper understanding of their phylogenetic relationships, species boundaries, and genetic structure.

*Primula mallophylla* Balf. F., belongs to the family Primulaceae, genus *Primula*, and section *Proliferae.* This plant vanished following its discovery in the late 19th century, only to be rediscovered by researchers after a span of 90 years, and is designated as Critically Endangered (CR) in the China Biodiversity Red List (Balfour, 1916; Wu et al., 2009; https://www.mee.gov.cn/xxgk2018/xxgk/xxgk01/202305/t20230522_1030745.html). Importantly, the vast majority of the plants in this section are distributed above 2km in altitude (Hu, 1990), which is a key taxon for studying environmental adaptations and evolutionary mechanisms. Therefore, the present study is the first to analyze the complete mitochondrial and chloroplast genomes of *P. mallophylla*, to comprehensively analyze its gene composition, structural features, repeat sequences, RNA editing, codon preferences, and selection pressure, exploring its evolutionary status and affinity in the *Primula* by combining phylogenetic analysis and collinear comparison. This study not only helps to clarify the taxonomic controversy and reveal the complex evolutionary process, but also offers a significant theoretical foundation for the conservation genetics of endangered plants.

## 2 Materials and methods

### 2.1 Plant materials, DNA extraction, and sequencing

Fresh young leaves of *P. mallophylla* were harvested from Chengkou County, Chongqing, China. Total DNA was extracted *via* the CTAB method (Doyle and Doyle, 1987), and its concentration and integrity were assessed using 0.7% agarose gel electrophoresis, NanoDrop One spectrophotometer (NanoDrop Technologies, Wilmington, DE, USA), and Qubit 3.0 fluorometer (Life Technologies, Carlsbad, CA, USA). A SMRTbell library with an insert size of 15-20 kb was then generated and sequenced on the PacBio Revio platform. Meanwhile, a paired-end library with an average insert size of 400 bp was generated and sequenced on the Illumina NovaSeq platform. Following quality control, adapter trimming, and the elimination of low-quality reads, 10 Gb HiFi long-reads and 4 Gb of Illumina short-reads were acquired.

### 2.2 Genome assembly and annotation

*De novo* assembly of the *P. mallophylla* mitochondrial genome was performed using Flye (v2.9) software with default parameters on HiFi long-reads (Kolmogorov et al., 2019). All assembled contigs were indexed using the makeblastdb program from BLAST (Altschul, 1997). Subsequently, the mitochondrial genome sequence of *Primula sikkimensis* (NC_082937.1-08293743.1) was used as a query to screen for contigs containing mitochondrial genome sequences *via* BLASTn (v2.13.0) (Chen et al., 2015), with parameter ‘-evalue 1e-10 -outfmt 6 -max_hsps 10 -word_size 7 -task blastn-short’. All long-reads were mapped to aligned contigs using Minimap2 (Li, 2018), and aligned reads were extracted and reassembled using Flye software. The assembly results were visualized using Bandage (v0.8.1) (Wick et al., 2015). The Protein-coding genes (PCGs) were annotated utilizing PMGA (Li et al., 2024a), referencing the mitochondrial genomes of *P. sikkimensis*, *Arabidopsis thaliana* (NC_037304), and *Liriodendron tulipifera* (NC_021152.1). The tRNA genes were annotated using tRNAscan-SE (v2.0.11) (Lowe and Eddy, 1997), whereas rRNA genes were annotated using BLASTn. All annotations underwent manual curation using Apollo (v1.11.8) (Lewis et al., 2002). The chloroplast genome was assembled *via* GetOrganelle (Jin et al., 2020), with the parameters ‘-R 15 -k 21,45,65,85,105 -F embplant_pt’. The assembly results were annotated with CPGAVAS2 (Shi et al., 2019), utilizing *Primula stenodonta* (NC_034677.1) as a reference. Annotation errors were manually modified using CPGView (Liu et al., 2023). Finally, both the mitochondrial and chloroplast genome maps were visualized using OGDRAW (Stephan et al., 2019).

### 2.3 Repeat sequence analysis

Repeat sequences of mitochondrial and chloroplast genomes, including simple sequence repeats (SSRs), tandem repeats, and dispersed repeats, were identified using the MISA webserver (v2.1) (Beier et al., 2017), the Tandem Repeats Finder (v4.09) (Benson, 1999), and the REPuter (Stefan et al., 2001), respectively. The outcomes were depicted with Microsoft Excel 2021 and the Circos package inside TBtools (Zhang et al., 2013; Chen et al., 2020).

### 2.4 RNA editing site prediction and codon usage bias analysis

RNA editing sites for mitochondrial genome PCGs were computationally predicted using Deepred-mt (Edera et al., 2021). The PCGs were extracted from mitochondrial and chloroplast genomes using Phylosuite (v1.1.16) (Zhang et al., 2020), and then relative synonymous codon usage (RSCU) values were calculated using MEGA (v7.0) (Kumar et al., 2016). The effective number of codons (ENC) values and GC content at the third synonymous codon position (GC3s) were calculated using CodonW (v1.4.4) (Grote et al., 2005). Results were visualized using ggplot2 (Wickham, 2011) in the R-package.

### 2.5 Intracellular gene transfer analysis

To detect potential sequence transfers between the mitochondrial and chloroplast genomes of *P. mallophylla*, pairwise sequence comparisons were performed using the BLASTn with an E-value threshold of 1e-5. Homologous fragments were then visualized using the Circos package inside TBtools.

### 2.6 Phylogenetic inference and divergence time estimation

A total of 16 species were selected for phylogenetic analyses based on mitochondrial and chloroplast genomes, of which 15 species belong to Ericales, with *Hydrangea chinensis* (Cornales) designated as the outgroup. The shared PCGs across mitochondrial and chloroplast genomes (15 and 59 genes, respectively) were extracted using PhyloSuite. After multiple sequence alignment with MAFFT (Katoh and Standley, 2013), correction using MACSE (Ranwez et al., 2018), and trimming with Gblocks (Talavera and Castresana, 2007), concatenated sequence matrices were generated for phylogenetic tree reconstruction. To evaluate phylogenetic signals under different evolutionary models, maximum likelihood (ML) and Bayesian inference (BI) analyses were conducted at both the nucleotide level and codon-partitioned level. ML analyses were performed using IQ-TREE (v1.6.12) (Nguyen et al., 2015), with the nucleotide model set as ‘-m MFP -B 1000 -alrt 1000’ and the codon-partitioned model as ‘-m MFP+MERGE -B 1000 -alrt 1000’. BI analyses were conducted in MrBayes v3.2.6 (Ronquist et al., 2012), with each dataset run for 2,000,000 generations, sampling every 1,000 generations, and discarding the initial 25% of samples as burn-in. Convergence was assessed using Tracer v1.7.2.

Additionally, phylogenetic analyses were performed based on complete chloroplast genomes of the 16 species to enhance resolution. To further resolve relationships within *Primula*, ML and BI trees were constructed based on 86 *Primula* species and two *Androsace* outgroup species using complete chloroplast genomes and 65 shared PCGs. To assess the consistency of phylogenetic signals, gene concordance factor (gCF) analyses were conducted on phylogenies inferred from chloroplast and mitochondrial PCGs using IQ-TREE under default settings, quantifying the concordance between individual gene trees and the overall species tree topology.

All published mitochondrial and chloroplast genomes of the species used in this analysis were retrieved from the NCBI database (accession numbers are listed in **Supplementary Tables S1, S2**). The best-fit nucleotide substitution models for each dataset were automatically selected by ModelFinder (Kalyaanamoorthy et al., 2017) implemented in IQ-TREE, and the specific PCGs datasets used for phylogenetic analyses are provided in **Supplementary Table S3**. Phylogenetic trees were visualized using iTOL v6 (Letunic and Bork, 2024).

Divergence times were estimated using the ML topology of the 16 species based on shared chloroplast PCGs. Three fossil calibration points were selected from Timetree5 (http://www.timetree.org/): *P. mallophylla*-*Aegiceras corniculatum* (34.6-54.5 Mya), *Rhododendron delavayi*-*Camellia sinensis* (82.8-106.0 Mya), and *C. sinensis*-*H. chinensis* (109.0–122.0 Mya). Divergence time estimation was conducted using the MCMCTree module of PAML v4.9j (Yang, 2007), with the trimmed concatenated nucleotide matrix as input. Analyses were run for 1,000,000 generations, sampling every 100 generations, with the initial 20% of samples discarded as burn-in. Two independent chains were run to verify consistency, and convergence was assessed using Tracer.

### 2.7 Selective pressure analysis

Based on the alignments of PCGs from the mitochondrial and chloroplast genomes of the 16 species, The KaKs_Calculator 2.0 (Wang et al., 2010) was used to calculate the nonsynonymous substitution rate (*Ka*), synonymous substitution rate (*Ks*), and the *Ka/Ks* ratio for each gene, providing a preliminary assessment of evolutionary rate variation among genes within the overall phylogenetic framework. R scripts were then used to generate density plots and boxplots.

Furthermore, based on the phylogenetic tree constructed from the mitochondrial and chloroplast PCGs of the 16 species, the codeml program in the PAML package was used to perform branch-site model analyses to detect potential signals of positive selection along specific lineages. The branch containing *P. mallophylla* was designated as the foreground branch, while all other branches were treated as background branches. Two models were fitted for comparison: Alternative model, which allows a proportion of sites on the foreground branch to be under positive selection (ω > 1), and the null model, which restricts all ω values to ≤ 1. The goodness of fit between the two models was compared using likelihood ratio tests (LRTs), and statistical significance was determined based on a chi-square distribution with one degree of freedom. Genes with *p* < 0.05 were considered to be under significant positive selection on the foreground branch.

In addition, to further explore the characteristics of positive selection within the genus *Primula*, the same analytical procedure was independently applied to the chloroplast PCGs dataset comprising 13 closely related *Primula* species and two outgroup species (**Supplementary Tables S2, S3**).

### 2.8 Genome structural comparison

The ten representative species of Ericales were selected for collinearity analysis of mitochondrial genomes. Conserved homologous sequences among these species were identified using BLASTn with parameters ‘-value 1e-5, - word_size 9, -gapopen 5, -gapextend 2, -reward 2, -penalty -3’. Based on the comparison results, the Multiple Synteny Plot was generated using MCscanX (Wang et al., 2012) for collinear blocks longer than 500 bp. Comparative analysis of the chloroplast genome structures of *P. mallophylla* and its four closely related species was conducted. The expansion and contraction of boundaries were examined and seen with IRscope (Amiryousefi et al., 2018), while sequence comparison was conducted using mVISTA (Frazer et al., 2004). To visually illustrate the rearrangements and structural differences of mitochondrial and chloroplast genomes, we performed whole-genome alignments using MUMmer v4 (Marçais et al., 2018) and generated gnuplot scripts with mummerplot, followed by the construction of dotplots.

## 3 Results

### 3.1 General features of organelle genomes

**Figure 1:**
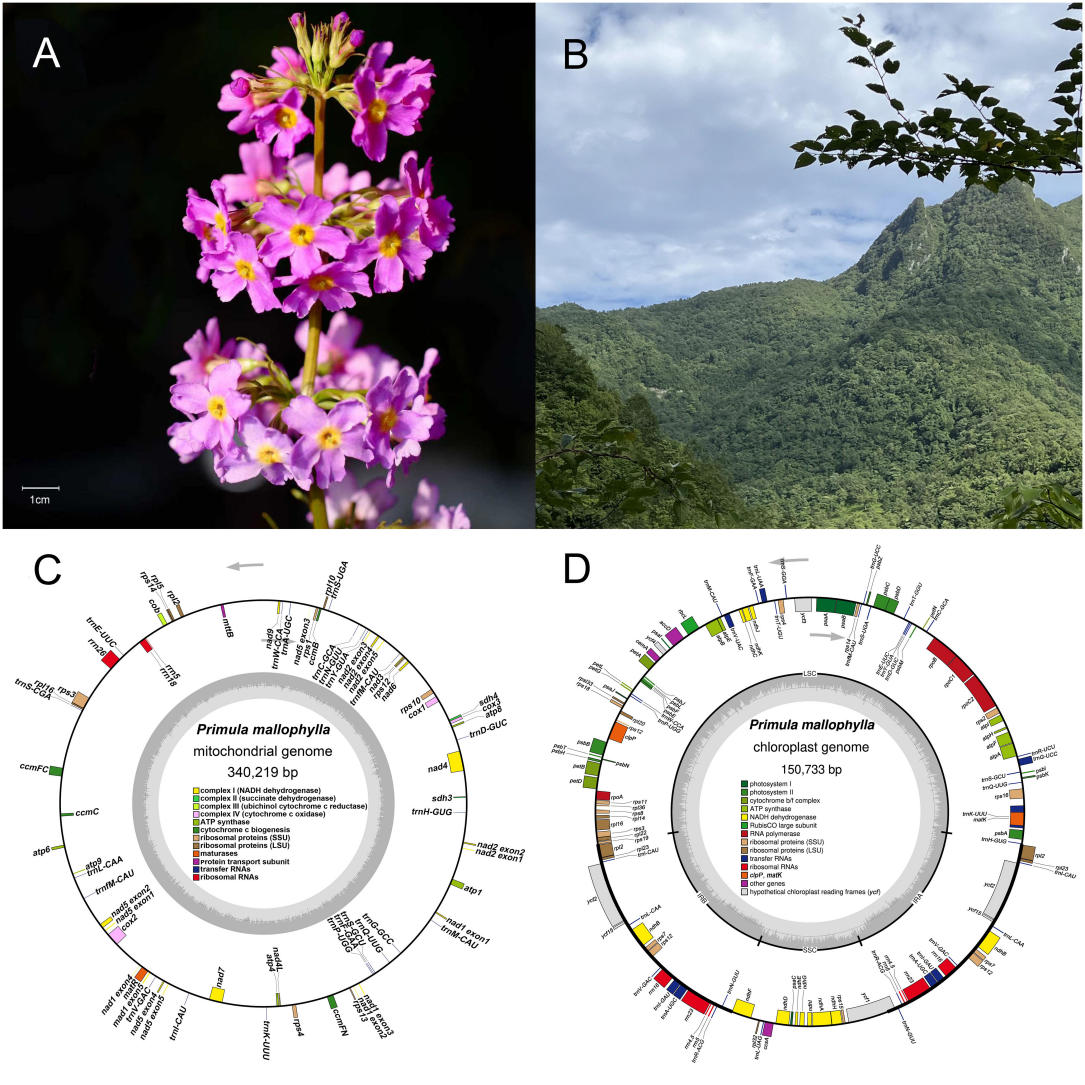
Morphology **(A)**, habitat **(B)**, and organelle genomes **(C, D)** of *P. mallophylla.* **(A)** shows a flowering individual, and both **(A, B)** were photographed in Houping Township, Chengkou County, Chongqing, China, by Prof. Hongping Deng. Genes in **(C, D)** are color-coded according to their functions, arrows indicate transcriptional direction, and the inner histogram represents GC content.

The high-quality mitochondrial and chloroplast genomes of *P. mallophylla* (**Figures 1A, B**) were sequenced, assembled, and annotated to identify variations and conservations (**Figures 1C, D**). The assembly results showed that each organelle genome of *P. mallophylla* was a closed-monocyclic structure (**Supplementary Figure S1**), with lengths of 340,219 bp and 150,733 bp, and GC contents of 45.57% and 37.09%, respectively. The chloroplast genome has a typical quadripartite structure, consisting of an 82,622 bp LSC region, a 17,743 bp SSC region, and two 25,184 bp IR regions.

In the mitochondrial genome, we annotated a total of 62 genes, including 37 unique PCGs, 22 tRNA genes, and 3 rRNA genes (**Supplementary Table S4**). Among the 37 unique PCGs, 24 were considered core genes, comprising five ATP synthase genes, nine NADH dehydrogenase genes, four cytochrome c biogenesis genes, three cytochrome c oxidase genes, one protein transport subunit gene, one maturase gene, and one cytochrome b gene. In the chloroplast genome, a total of 175 genes were annotated, including 130 PCGs, 37 tRNA genes, and 8 rRNA genes.

### 3.2 Repeat sequences analysis

**Figure 2:**
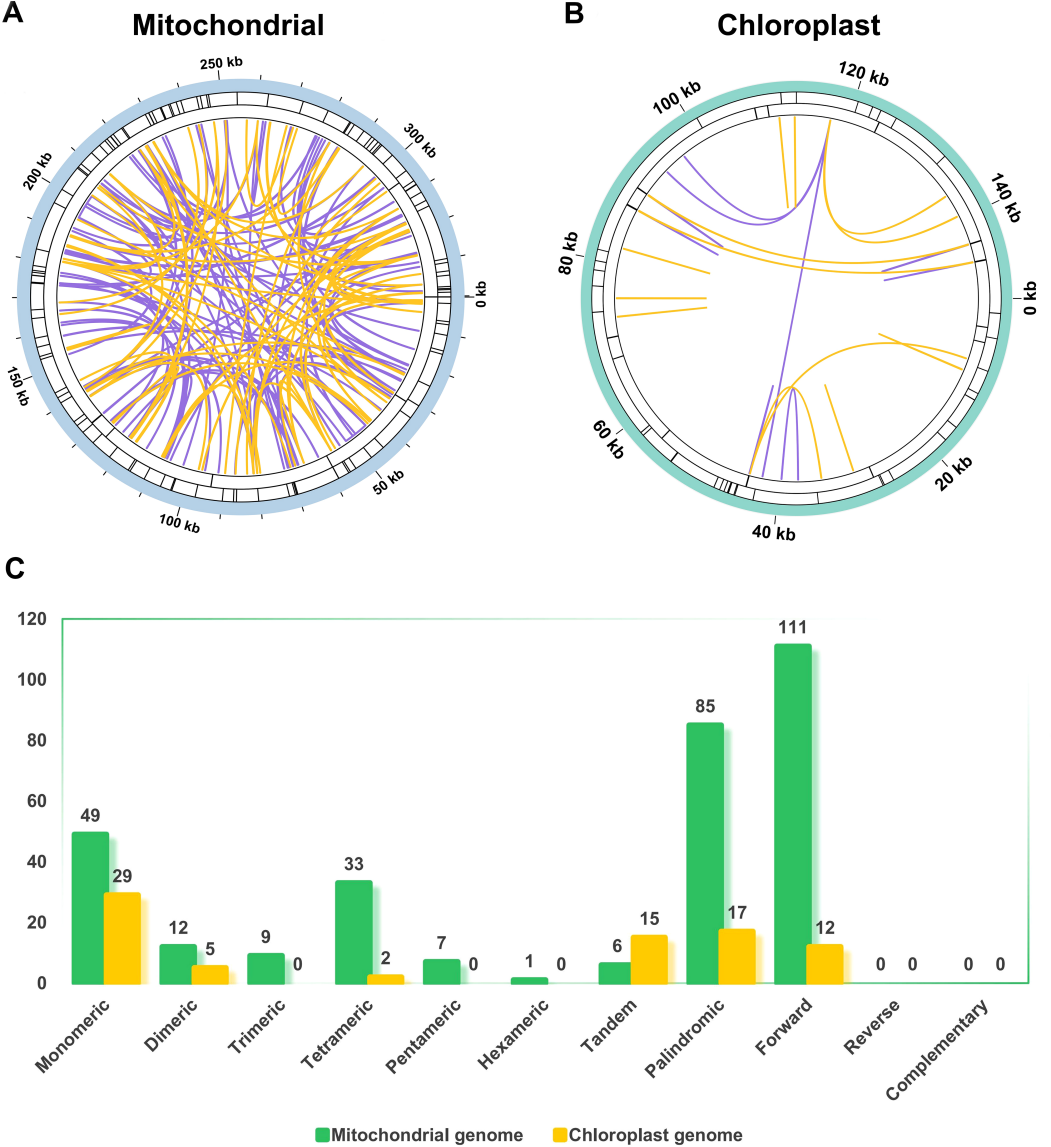
Analysis of repeat sequences in two organelle genomes of *P. mallophylla*. **(A, B)** depict the spatial distribution of repeat sequences inside the mitochondrial and chloroplast genomes, respectively. In the innermost circle, pairs of dispersed repeats are connected by colored lines, with palindromic repeats shown in yellow and forward repeats shown in purple. The black line segments on the middle and outermost circles denote the locations and lengths of tandem repeats and SSRs, respectively. **(C)** presents a bar chart summarizing the counts of each type of repeat sequence in the organelle genomes.

The mitochondrial genome and chloroplast genome of *P. mallophylla* differed significantly in the type and distribution of repeat sequences (**Figure 2**). The mitochondrial genome was more abundant in repeat sequences, with a total of 111 SSRs, 6 tandem repeats, and 196 dispersed repeats identified, which were widely distributed in various regions of the genome. Among the SSRs, monomeric repeats were dominant, particularly A/T, which accounted for 44.14%. The 6 tandem repeats were all located in the intergenic region, and the repeat units ranged from 11–57 bp. The dispersed repeats included only palindromic and forward repeats, accounting for 43.37% and 56.63%, respectively. However, most of the repeat units were between 30 bp and 100 bp in length, and only 13 repeat units were longer than 100 bp. The longest dispersed repeat was a 7,613 bp forward repeat, which could be a potential region for genomic rearrangement. In contrast, the chloroplast genome has relatively conserved repeat sequences. There were 36 SSRs, 15 tandem repeats, and 29 dispersed repeats in the chloroplast genome. The SSRs consisted of 29 monomeric repeats (all A/T), 5 dimeric, and 2 tetrameric, which were predominantly distributed in the LSC region. In contrast to the mitochondrial genome, the chloroplast genome contained more palindromic repeats than forward repeats, and the repeat units were all within 30–60 bp in length.

### 3.3 Prediction of RNA editing events

**Figure 3:**
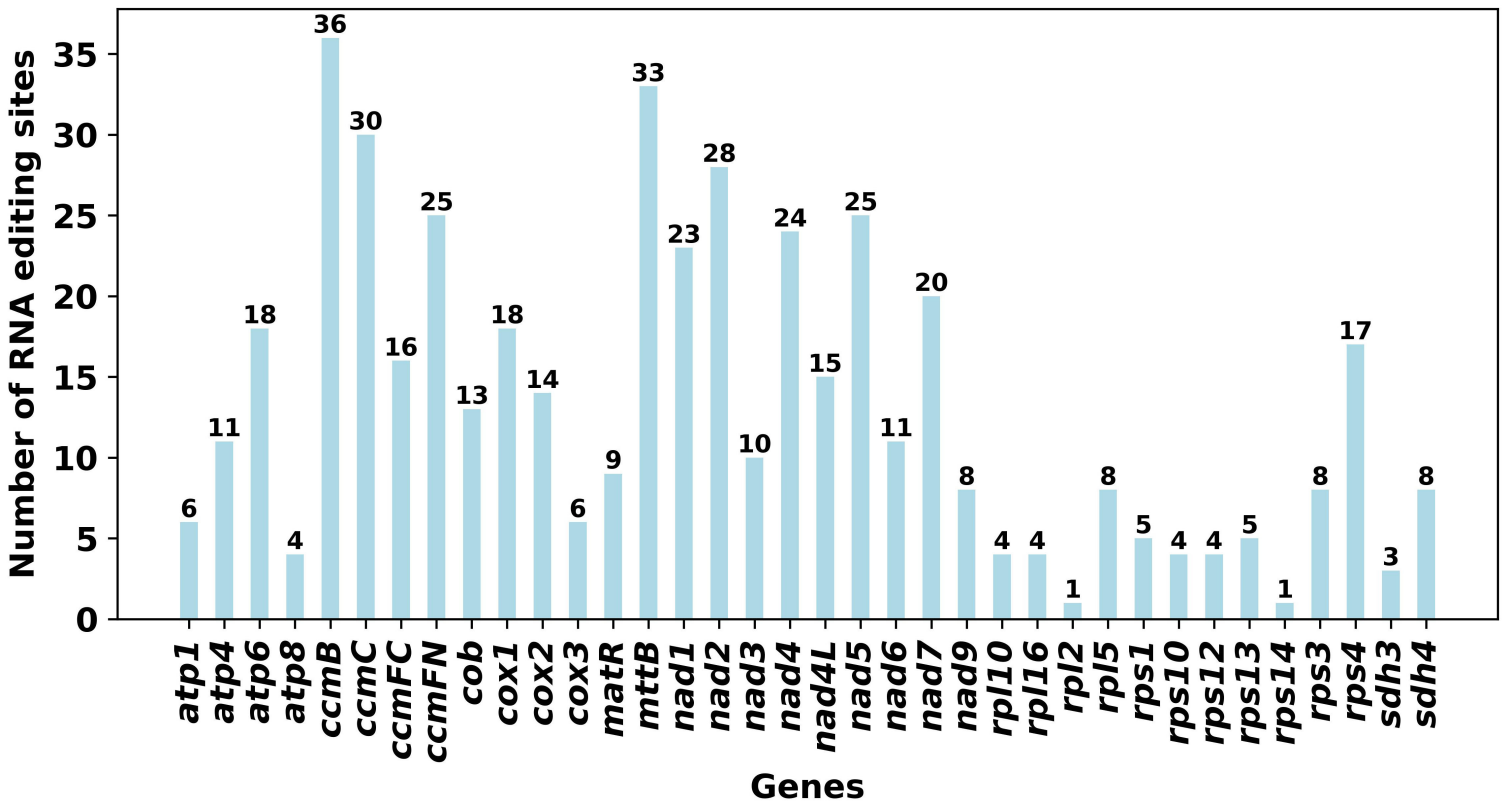
Identification of RNA editing sites for PCGs in the mitochondrial genome of *P. mallophylla*.

We used 37 unique PCGs from the mitochondrial genome of *P. mallophylla* to predict RNA editing events, identifying a total of 475 predicted sites (**Figure 3**). Notably, the *ccmB* gene demonstrated the greatest frequency of RNA editing events, with 37 sites. In contrast, the *rps14* and *rpl2* genes exhibited less RNA editing activity, possessing only one editing site each, suggesting lower variability in these genes. Further analysis showed that 210 RNA editing sites (44.21%) changed from hydrophilic to hydrophobic amino acids, 155 sites (32.63%) from hydrophobic-hydrophobic, 67 sites (14.11%) from hydrophilic-hydrophilic, 36 sites (7.58%) from hydrophobic-hydrophilic, and 7 sites (1.47%) from hydrophilic-stop (**Supplementary Table S5**). In addition, 213 sites (44.84%) were changed to leucine, indicating leucine propensity.

### 3.4 Condon usage bias analysis

**Figure 4:**
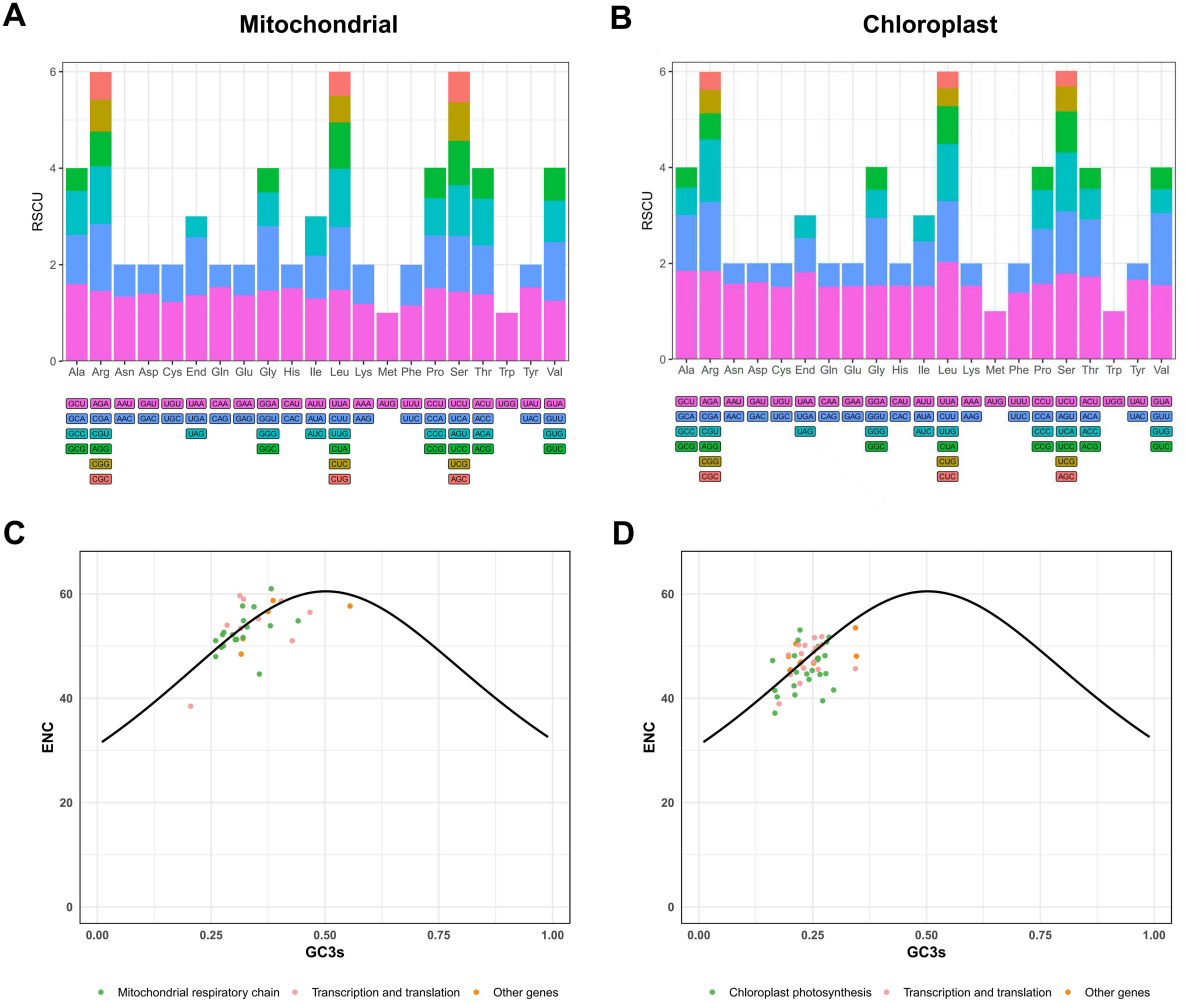
Codon usage characteristics of PCGs in the organelle genomes of *P. mallophylla*. **(A, B)** depict the distribution of RSCU values in the mitochondrial and chloroplast genomes, respectively. The horizontal axis represents 20 amino acids and stop codon (End), while the vertical axis indicates RSCU values for the different codons. **(C, D)** display ENC-GC3s plots for the mitochondrial and chloroplast genomes, respectively. The solid curve represents the expected ENC values under the assumption that codon usage bias is solely driven by GC3s composition. In both plots, green dots represent genes involved in the respiratory chain **(C)** or photosynthesis **(D)**, pink dots denote genes related to transcription and translation, and orange dots correspond to other genes.

The codon usage frequencies in each organelle genome of *P. mallophylla* were approximately the same, but with some differences (**Figures 4A, B**). In both the mitochondrial and chloroplast genomes, the RSCU values for the majority of codons, excluding AUG (the start codon for methionine) and UGG (which encodes tryptophan), were either above or below 1. This suggests a generalized codon usage bias for the PCGs in each organelle genome. In the mitochondrial genome, 29 codons exhibited RSCU values over 1, with alanine showing the greatest preference for the GCU codon (RSCU = 1.6). In the chloroplast genome, 29 codons exhibited RSCU values over 1, with the UUA codon for leucine showing the strongest usage bias, having an RSCU value of 2.03 (**Supplementary Table S6**). In addition to this, of these codons, only 2 codons in the mitochondrial and 1 codon in the chloroplast genome ended with C/G endings, and the rest of the codons were A/U endings, showing a strong preference for A/U usage.

ENC-GC3s analysis can further help understand the underlying drivers behind codon usage (Li et al., 2023). In each organelle of *P. mallophylla*, most of the PCGs were distributed on or near the standard curve (**Figures 4C, D**). In mitochondria, *rps1*, *rps13*, *and sdh3* were farthest from the standard curve, whereas in chloroplasts, *atpF*, *psbA*, *and rps4* were farthest.

### 3.5 Homologous sequences analysis between two orangelle genomes

**Figure 5:**
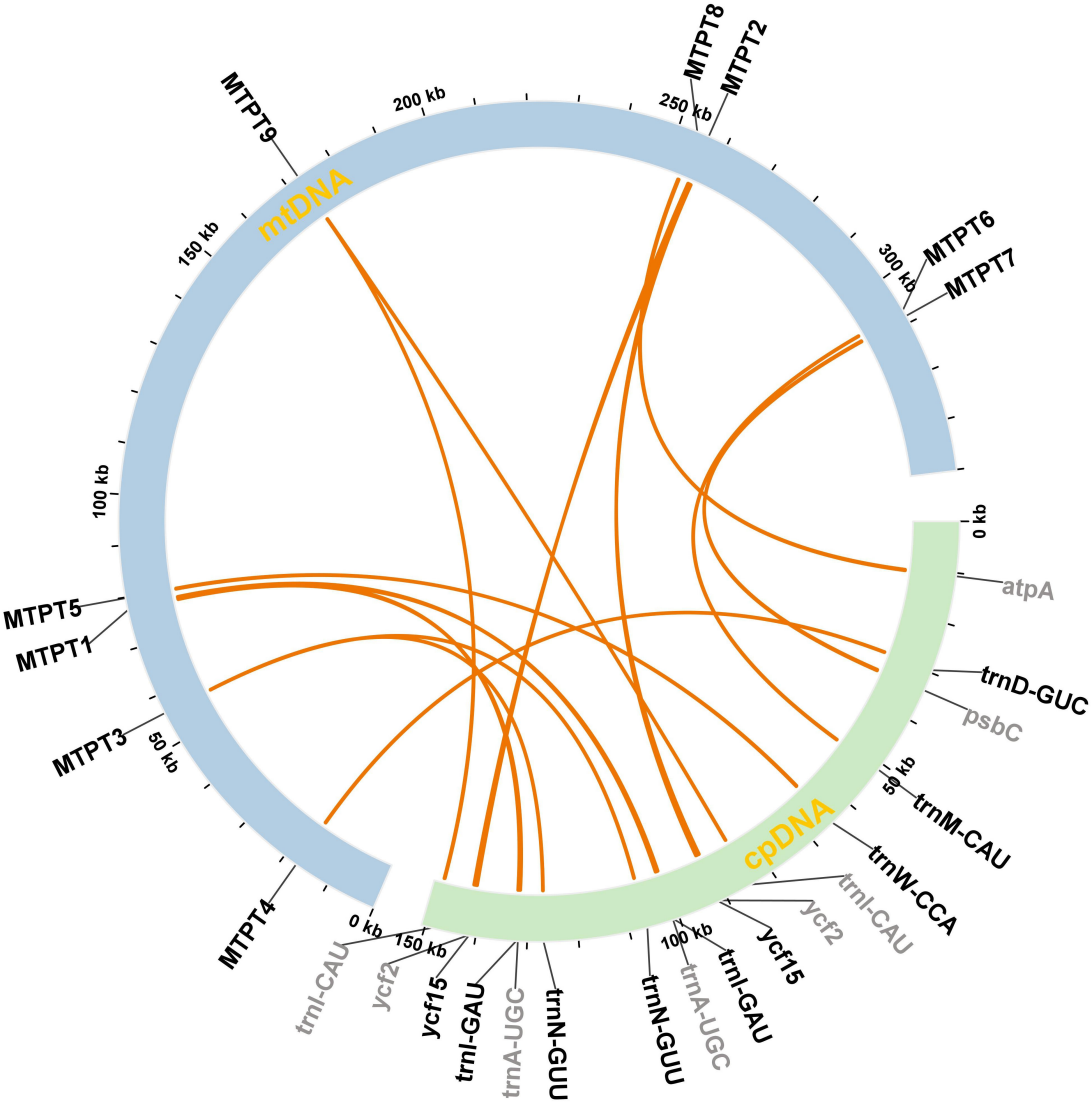
Intracellular genome transfer in *P. mallophylla*. The mitochondrial genome is illustrated in blue, the chloroplast genome in green, and the orange arcs within the circular ring represent homologous segments shared by both genomes. Complete genes are labeled in black on the outer circle, whereas partial genes are shown in gray.

Horizontal gene transfer (HGT) is a common phenomenon in angiosperms. To investigate potential intracellular transfers, we examined the migration of chloroplast-derived sequences into the mitochondrial genome of *P. mallophylla* (**Figure 5**). A total of six mitochondrial plastid DNA (MTPTs) were identified, spanning 2,028 bp in total. These MTPTs contained six complete genes, including 1 PCGs (*ycf*15) and 5 tRNA-encoding genes (*trnD-GUC*, *trnI-CAU*, *trnM-CAU*, *trnN-GUU*, *trnW-CCA*) (**Supplementary Table S7**). Among these transfer segments, the longest homologous fragment detected was MTPT2, which was 728 bp in length and contained two genes, the complete gene *ycf15* and part of the gene *ycf2*.

### 3.6 Phylogenetic relationships and divergence time estimation

**Figure 6:**
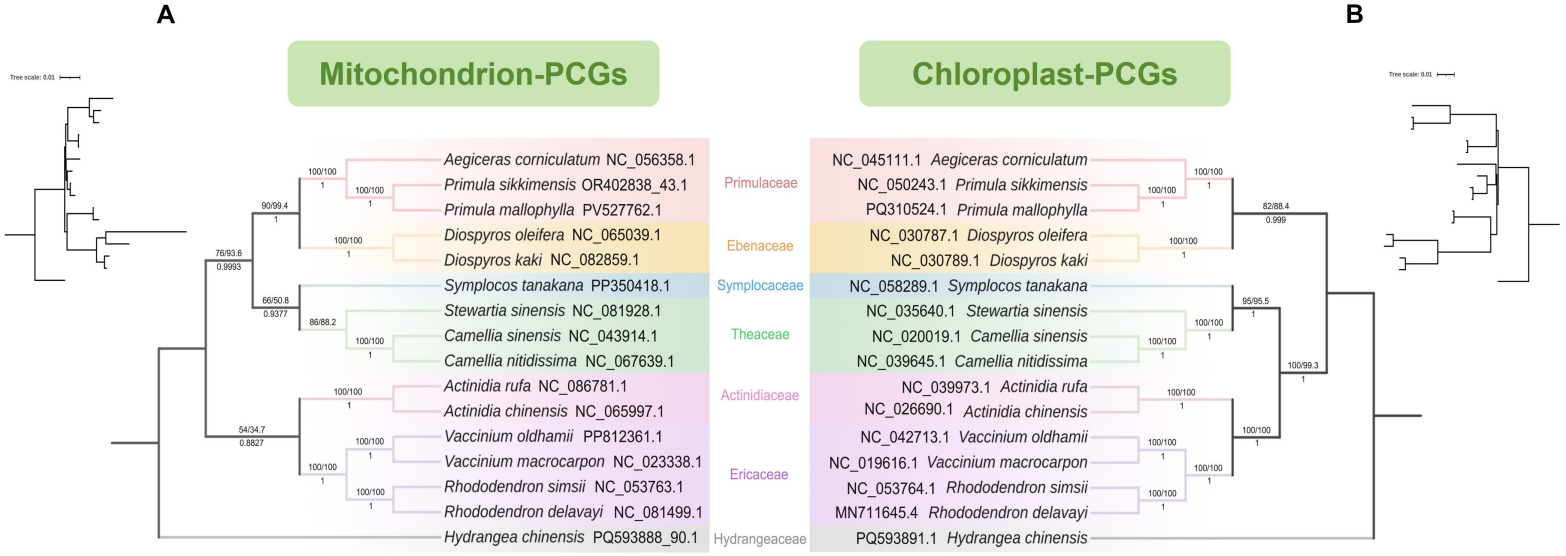
Maximum likelihood (ML) and Bayesian inference (BI) phylogenetic trees constructed based on shared PCGs from the mitochondrial genome **(A)** and chloroplast genome **(B)** of 16 species. Branches show bootstrap/SH-aLRT support values above and Bayesian posterior probabilities below. In panel B, nodes indicate divergence times with 95% confidence intervals. NCBI accession numbers for each species are provided.

Phylogenetic trees were constructed based on the shared PCGs of mitochondrial and chloroplast genomes, including 15 species of Ericales and one outgroup. The results showed that (**Figure 6**; **Supplementary Figures S2A, S2B**), for both the DNA sequence and codon-partitioned datasets, the topologies of the ML and BI trees were fully congruent, indicating a high consistency of phylogenetic signal within each dataset. The results showed that both mitochondrial and chloroplast PCG-based phylogenetic trees consistently indicate a close relationship between Primulaceae and Ebenaceae. However, the phylogenetic trees based on mitochondrial and chloroplast PCGs showed some differences in certain clades. In the mitochondrial PCG tree, clade I (Primulaceae and Ebenaceae) was closer to clade II (Symplocaceae and Theaceae) with low support (**Figure 6A**), whereas in the chloroplast PCG tree, clade II was closer to clade III (Actinidiaceae and Ericaceae) (**Figure 6B**). Further analyses based on complete chloroplast genomes showed that the ML tree placed clade II closer to clade III (**Supplementary Figure S2C**), while the BI tree grouped all families except Primulaceae into one clade, with a low posterior probability of 0.7505 (**Supplementary Figure S2D**). To assess the congruence of phylogenetic signals, we conducted gCF analysis on trees constructed from mitochondrial and chloroplast genome PCGs. The results showed that the gCF values for clade I were 33.33% and 22.03% in the mitochondrial and chloroplast genomes, respectively. In the mitochondrial genome, the gCF value between clade I and clade II was 20%, whereas in the chloroplast genome, the gCF value between clade II and clade III was 16.95%. Overall, the phylogenetic signals of different organelle genomes showed some differences, but both supported the primary familial relationships within Ericales. Based on this 16-species phylogeny, the divergence time between *P. mallophylla* and *P. sikkimensis* was estimated at approximately 2.13 Mya, and the divergence between *Primula* and *Aegiceras* at approximately 42.99 Mya (**Figure 6B**).

**Figure 7:**
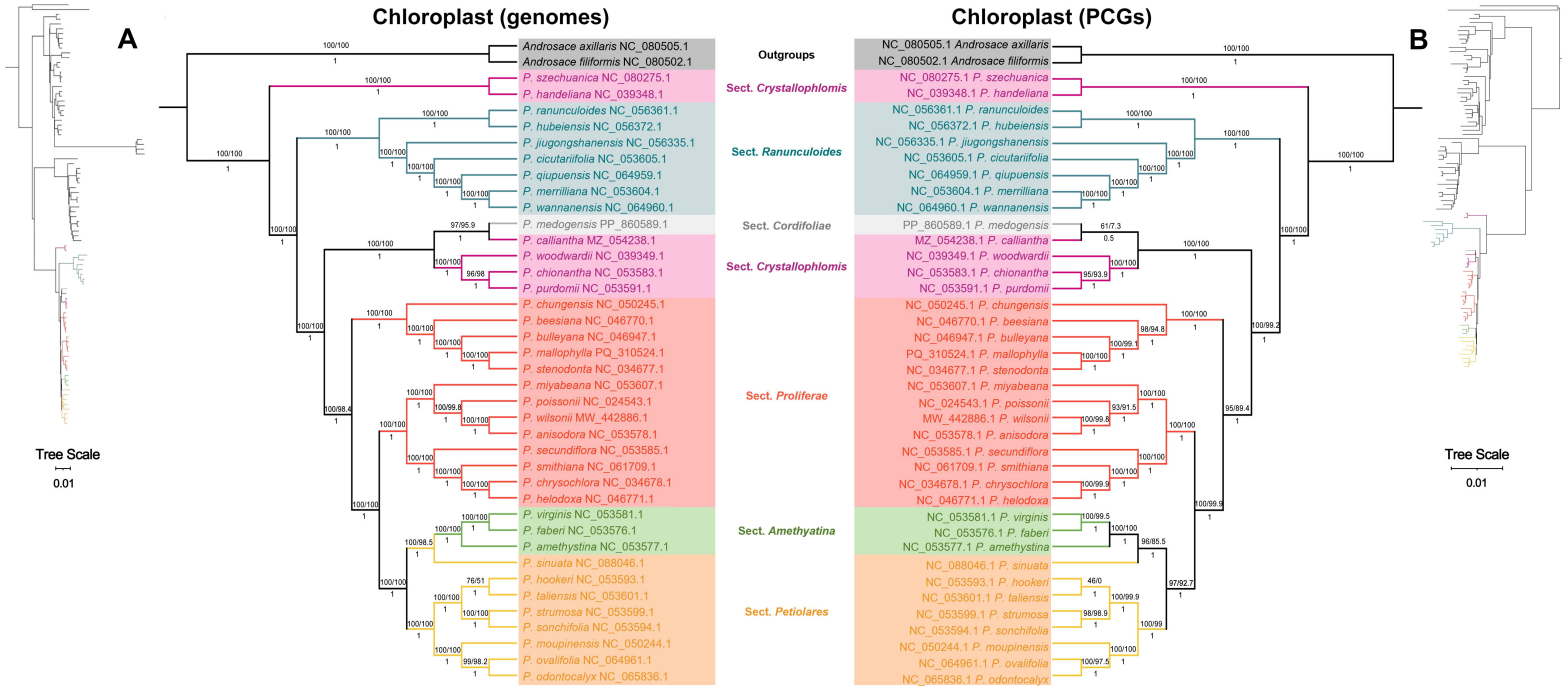
Maximum likelihood (ML) and Bayesian inference (BI) phylogenetic trees constructed from the chloroplast genomes **(A)** and shared PCGs **(B)** of 88 species. Branches show bootstrap/SH-aLRT support values above and Bayesian posterior probabilities below. NCBI accession numbers for each species are provided.

Further ML and BI trees were constructed for the complete chloroplast genomes (**Figure 7A**) and shared PCGs (**Figure 7B**) of 86 species in *Primula* to clarify the evolutionary relationships of this genus. The results showed that 88 species of *Primula* were clustered into three large clades, and the trees constructed based on chloroplast genomes and the PCGs were essentially consistent with each other. The sect. *Proliferae* was divided into 2 groups, of which *P. mallophylla* was clustered with *P. stenodonta*, *P. bulleyana*, *P. beesiana*, *P. chungensis*, and was closest related to *P. stenodonta*. In addition to the sect. *Proliferae*, the sect. *Crystallophlomis* and *Petiolares* were also not a monophyletic group.

### 3.7 Selective pressure on PCGs in organelle genomes of *Primula*

To investigate the evolutionary patterns of PCGs in different organelle genomes, *Ka/Ks* analyses were conducted on mitochondrial and chloroplast PCGs based on a phylogeny of 16 species (15 Ericales species and one outgroup) (**Supplementary Figure S3**). Chloroplast genes exhibited higher *Ks* values than mitochondrial genes, indicating faster accumulation of neutral mutations. Most genes in both organelles had *Ka/Ks* < 1, suggesting strong purifying selection. In the mitochondrial genome, *atp4*, *ccmB*, and *ccmFN* showed evidence of positive selection, whereas no chloroplast genes were under significant positive selection. Branch-site analyses revealed that, when *P. mallophylla* was set as the foreground branch, no significantly selected genes were detected in either organelle. When *Primula* was the foreground branch, *rpl22* and *rbcL* in the chloroplast genome were under significant positive selection (*p* < 0.05), indicating potential adaptive evolution (**Supplementary Table S8**).

Further analyses of 13 *Primula* species plus two outgroup showed that chloroplast PCGs were overall under strong purifying selection (**Supplementary Figure S4**), but in *P. mallophylla*, *rpl2* and *ndhB* were significantly positively selected (*p* < 0.05), suggesting roles in species-specific adaptation (**Supplementary Table S8**).

### 3.8 Comparative analysis of organelle genome structure

**Figure 8:**
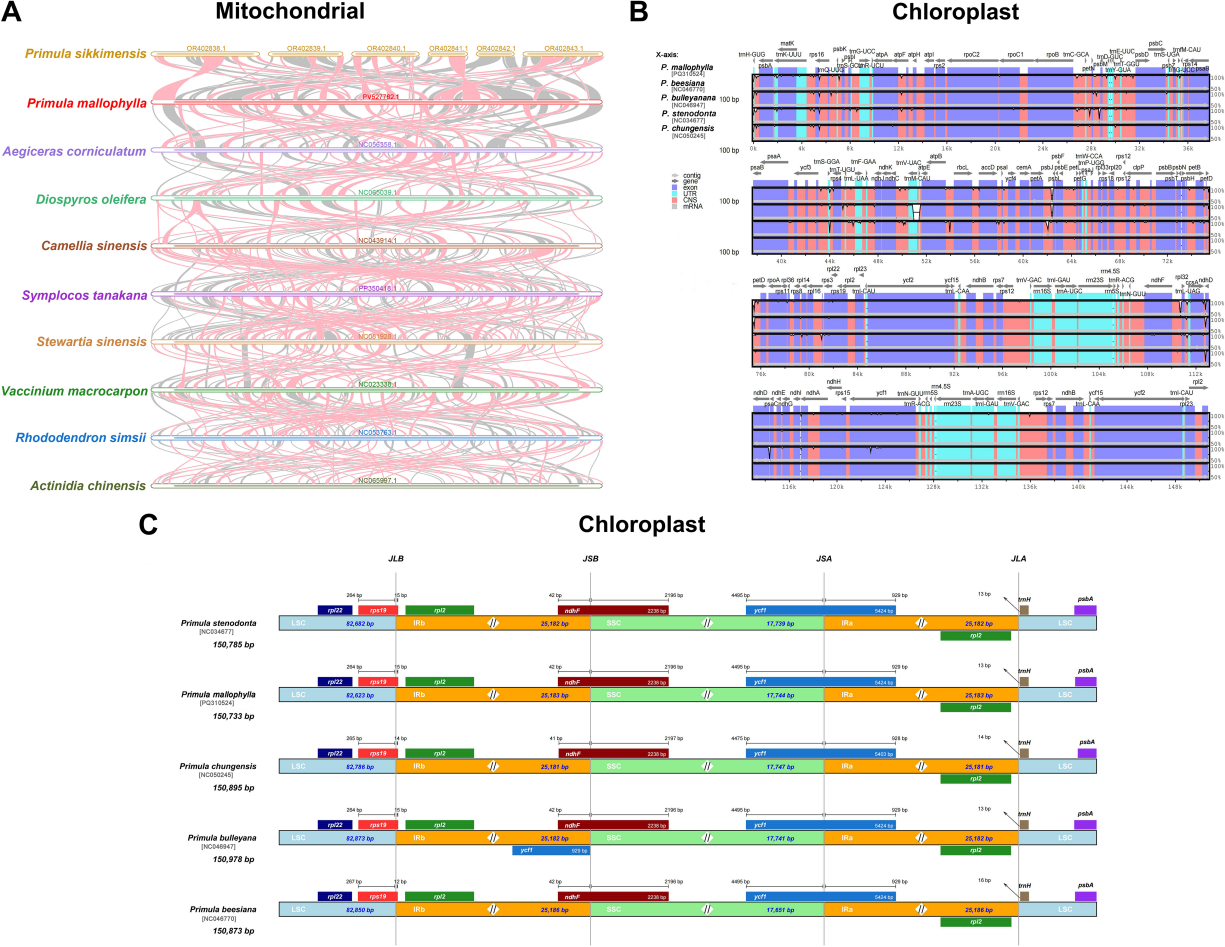
Comparative analysis of the structural characteristics of organelle genomes. **(A)** Collinearity analysis of the mitochondrial genomes from 10 representative species of Ericales, with red arcs indicating inversion regions and gray areas representing homologous regions. **(B)** mVISTA analysis of the chloroplast genomes from five *Primula* species, where exons, untranslated regions (UTRs), conserved noncoding sequences (CNS), and mRNA are distinctly color-coded. Gray arrows indicate the direction of gene transcription, and the vertical axis denotes the proportion of sequence identity ranging from 50% to 100%. **(C)** Visualization of the chloroplast IR boundaries in five *Primula* species. JLB, JSB, JSA, and JLA represent the junctions between the LSC and IRb, SSC and IRb, SSC and IRa, and LSC and IRa, respectively.

Comparison of mitochondrial genome homologous fragments from 10 representative species of the Ericales showed that the length of their collinear blocks (>500bp) was short (**Figure 8A**). A total of 103 homologous collinear blocks in length were identified among the *P. mallophylla*, *P. sikkimensis*, and *Aegiceras corniculatum*, all belonging to the Primulaceae family. The longest collinear block up to 9,987bp between *P. mallophylla* and chromosome 2 of *P. sikkimensis*, which are sister species in the phylogenetic tree. This indicated that the mitochondrial genome of *P. mallophylla* underwent rearrangement with closely related species and was extremely non-conservative in the structure.

Phylogenetic analysis of the chloroplast genome indicated that *P. stenodonta*, *P. bulleyana*, *P. beesiana*, and *P. chungensis* are most closely related to *P. mallophylla*. Consequently, we visualized and compared these five species' chloroplast genomes to reveal their genome structure variation characteristics more comprehensively (**Figures 8B, C**). The result showed that the coding regions exhibit greater conservation than non-coding regions. The majority of the observed changes were located in non-coding sequence regions (**Figure 8B**). Then, we compared the IR boundaries of five *Primula* species. The results indicated that IR regions exhibited greater conservation than the LSC and SSC regions, and all five species were found to have the same genes at the boundaries, but with slightly different sizes (**Figure 8C**). The mitochondrial and chloroplast genome dotplots of *P. mallophylla* and its closely related species are shown in **Supplementary Figure S5**.

## 4 Discussion

### 4.1 Genomic features of organelles of *P. mallophylla*

In this study, we successfully assembled high-quality mitochondrial and chloroplast genomes of the endangered species *P. mallophylla*. Due to the advantages of long segments and high accuracy of HiFi long-reads, it is able to span complete replicates for continuous and accurate assembly. Therefore, our assembled mitochondrial genomes are reliable and bring an extremely important reference to the study of *Primula*, which is currently very scarce. In comparison to the mitochondrial genomes of three *Primula* species constructed from second-generation data (Wei et al., 2022) and the unpublished mitochondrial genome of *P. sikkimensis* (OR402838.1- OR402843.1), the mitochondrial genomes of *P. mallophylla* were essentially the same as them in terms of length (340, 219 bp) and GC content (45.57%). Unlike the six chromosomes of *P. sikkimensis*, *P. mallophylla* exhibits the typical master circular molecules consistent with many higher plants. In addition, collinear analysis showed that *P. mallophylla* had more collinear blocks with *P. sikkimensis*, but most of them underwent rearrangements. Differences in mitochondrial genome structure between these two species may be a key driver of species differentiation and adaptation to different environmental stresses. In the common ancestor of angiosperms, there are typically 41 conserved and stable PCGs in mitochondrial genomes (Mower et al., 2012). Of these, 24 core PCGs are possessed in almost all species, leaving 17 variable genes that vary from species (Skippington et al., 2015). The mitochondrial genome of *P. mallophylla* has 37 PCGs, with 24 core genes. However, the ribosomal small subunit genes, *rps2*, *rps7*, *rps11*, and *rps19* are missing among the variable genes. Except for *rps19*, *P. mallophylla* is in agreement with *P. palinuri* and *P. smithiana* (Wei et al., 2022). The *rps2* gene has been reported to be lost from the mitochondrial genomes of most dicotyledons and transferred to the nucleus (Perrotta et al., 2002). However, it is still retained in a few dicotyledons, such as *Nelumbo nucifera* (Gui et al., 2016). The loss of the *rps7*, *rps11*, and *rps19* is prevalent in angiosperms (Adams et al., 2002). Overall, the *P. mallophylla* mitochondrial genome is highly conserved during evolution.

The chloroplast genome of *P. mallophylla* exhibits a characteristic quadripartite structure, conserved in accordance with other *Primula* species (Ren et al., 2018; Xie et al., 2023). Because chloroplasts carry key functions of photosynthesis and basic metabolism in plants, they are driven by strong purifying selection so that they are usually highly conserved structurally (Jansen and Ruhlman, 2012). According to the phylogenetic results, *P. mallophylla* is closely related to *P. stenodonta*, *P. bulleyana*, *P. beesiana*, and *P. chungensis*. Comparative structural analyses indicated that the chloroplast genome structures of these five species were markedly similar and likely diverged within a relatively short time. In contrast, the chloroplast genomes of species in the other group of the sect. *Poliferea* are relatively more structurally diverse and may have undergone more evolutionary events, including genome rearrangements, expansions, or contractions (Xie et al., 2023).

In the organelle genomes of *P. mallophylla*, we detected a large number of repeat sequences, which are crucial for maintaining organelle genome structure and mediating genome reorganization. We found that the repeat sequences in mitochondria far exceeded those in chloroplasts. The mitochondrial genome has evolved to undergo more complex gene rearrangements, losses, and transfers, which together drive the high diversity and complexity of its structure (Wynn and Christensen, 2019). In contrast, the chloroplast genome is more conserved, with less accumulation of repeat sequences. In the mitochondrial genome, repeat sequences are mainly dominated by dispersed repeats, whereas in the chloroplast genome, they are dominated by SSRs, which is related to the differences in genome structure and function between the two.

In addition to the important exchange of genetic material that occurs between the organelle genomes and the nuclear genome, such exchanges are very common among organelles, especially chloroplast-to-mitochondrial sequence transfer (MTPT), which is crucial for plant growth and adaptive evolution (Thorsness and Weber, 1996; Cusimano and Wicke, 2016; Cheng et al., 2021). In *P. mallophylla*, we identified 9 MTPTs with a cumulative length of 2028 bp, constituting 0.60 % of the total length of the mitochondrial genome, which aligns with the structural characteristics of other *Primula* plants (Xie et al., 2023). Among these homologous fragments, the vast majority are complete or partial fragments of chloroplast *trn* genes (*trnN-GUU*, *trnD-GUC*, *trnW-CCA*, *trnM-CAU*, *trnI-GAU*, *trnA-UGC*, *trnI-GAU*). Research indicates that mitochondrial tRNAs are derived not only from ancestral mitochondria but also from chloroplasts through horizontal gene transfer (HGT) (Sprinzl and Vassilenko, 2005; Rice et al., 2013). Although the sequence lengths of the respective MTPTs in *P. mallophylla* are short, it is also well demonstrated that these chloroplast-derived tRNAs play an essential role in maintaining important mitochondrial functions (Kanno et al., 1997; Menand et al., 1998).

### 4.2 Expression patterns and adaptive evolution of organelle genomes in *P. mallophylla*

In plant organelle genomes, the process of gene expression is uniquely regulated, especially in terms of RNA editing at the post-transcriptional stage and codon usage preferences at the translational stage, which are important for the stability and adaptation of organelle function.

In plant mitochondria, post-transcriptional mRNAs often undergo C-U RNA editing, allowing the edited protein sequences to more closely resemble homologous proteins from other species, enhancing sequence conservation across species and further optimizing the efficiency of gene expression within mitochondria (Covello and Gray, 1989). In *P. mallophylla*, we predicted 475 editing sites for C-U. Among them, *ccmB* and *mttB* have the highest editing frequencies, which are also high in *Primula* (Wei et al., 2022), *Dendrobium* (Wang et al., 2024b), *Solanum muricatum* (Li et al., 2024b) and other species, and are usually the two most frequently edited genes, which may be related to the maintenance of mitochondrial respiratory chain policy operation and energy metabolism. Furthermore, we discovered that 44.21% of amino acids transitioned from hydrophilic to hydrophobic following RNA editing, enhancing the protein's folding and functionality (Yura and Go, 2008). It is noteworthy that the RNA editing results in this study were predicted using the Deepred-mt model. This approach efficiently identifies potential C-to-U editing sites in mitochondrial genes and provides valuable reference information. However, as model-based predictions are influenced by algorithmic and parameter settings, some false positives may exist. Therefore, the present results mainly represent the putative mitochondrial RNA editing characteristics of *P. mallophylla*.

The preference of amino acids for synonymous codon usage during the translation step is prevalent across various prokaryotic and eukaryotic organisms (Zhang et al., 2007). Codon use bias is shaped by mutational pressure, natural selection, and genetic drift, significantly contributing to the adaptive evolution of plants (Parvathy et al., 2022; Lyu et al., 2024). Eeach organelle genome of *P. mallophylla* has 29 codons with RSCU values over 1, with the predominant majority terminating in A/U, aligning with the preference shown in most plants, indicating a degree of consistency in codon usage among plant species (Liu et al., 2024a; Wang et al., 2024b). ENC-GC3s can further determine whether codon usage preferences are mainly influenced by mutational pressure or natural selection (Li et al., 2023; Wright, 1990). The ENC-GC3s theoretical curve represents the trend of the ENC with the GC content of GC3s driven by GC mutation preference only. If the measured values of ENC are significantly lower than the theoretical values, it suggests that natural selection may have a potential influence. The *rps1*, *rps13*, and *sdh3* genes in mitochondria and the *atpF*, *psbA*, and *rps4* genes in chloroplasts exhibit significant differences from theoretical values, which may be subject to strong selection due to adaptation to the environment.

In studies of adaptive evolution in organelle genomes, *Ka* and *Ks* are commonly used to evaluate gene mutation accumulation and the strength of natural selection. The *Ka/Ks* ratio can reveal whether a gene is subject to purifying selection, positive selection, or neutral evolution. Our analysis of the evolutionary rates and selective pressures of PCGs in the organelle genomes of Ericales species found that the chloroplast genome has higher *Ks* values than the mitochondrial genome, further confirming that plant mitochondrial genes evolve more slowly than chloroplast genes (Drouin et al., 2008). However, the overall *Ka/Ks* ratio of the chloroplast is lower than that of the mitochondrion, indicating that although chloroplast genes are more prone to mutations, their evolution is under stricter purifying selection and exhibits greater functional conservation. This difference may arise from distinctions between the two organelles in replication frequency, repair mechanisms, and the intensity of functional constraints (Christensen, 2013; Smith and Keeling, 2015). Overall, mitochondrial and chloroplast genes are under strong purifying selection due to their critical roles in core functions such as energy metabolism and photosynthesis. Further branch-site analyses were conducted to identify branch-specific positive selection. When the genus *Primula* was set as the foreground branch, *rpl22* and *rbcL* in the chloroplast were detected as positively selected genes (*p* < 0.05). In an independent analysis of 13 *Primula* species plus two outgroup, chloroplast PCGs were generally under purifying selection, but *rpl2* and *ndhB* were identified as positively selected in *P. mallophylla* (*p* < 0.05). These four genes are closely associated with plant adaptation to alpine stresses such as low temperature, high light, and UV-B radiation (Nyamgerel et al., 2024; Zhang et al., 2024; Lu et al., 2025). *rbcL*, encoding the large subunit of Rubisco, may help maintain photosynthetic efficiency under high-altitude light and temperature conditions; *ndhB*, involved in the chloroplast NADH dehydrogenase complex, may facilitate cyclic electron flow in photosystem I, mitigating oxidative damage under high light; *rpl2* and *rpl22*, both ribosomal large subunit proteins, may reflect adaptive regulation of protein translation efficiency or stability in alpine environments.

### 4.3 Phylogenetic inference based on different organelle genomes

Mitochondrial and chloroplast genomes are usually matrilineally inherited and conserved, providing clear genetic signals that facilitate phylogenetic analysis. Due to the limited mitochondrial genomes in the Primulaceae, we reconstructed the phylogeny of related families of Ericales utilizing the conserved PCGs of the mitochondrial and chloroplast genomes. Phylogenetic analyses using different datasets and methods consistently support a stable placement of Primulaceae, generally showing a close relationship with Ebenaceae. Some topological differences were observed, with PCG-based trees clustering Primulaceae with Ebenaceae, while the BI tree based on complete chloroplast genomes grouped all other Ericales families together, consistent with chromosome-level genome analyses (Yang et al., 2022; Feng et al., 2021). These discrepancies likely reflect differences in evolutionary rates, gene rearrangements, and limited phylogenetic signal in individual PCGs, yet the overall results confirm the reliable phylogenetic position of Primulaceae.

The chloroplast genomes of 88 species were used to further explore the phylogenetic relationships of the *Primula* in depth. The ML and BI trees constructed on the basis of the whole chloroplast genome and conserved PCGs were basically consistent with each other, dividing the *Primula* into three major clades, which is in agreement with the results of a previous study (Xie et al., 2023). Focusing on the clades in which sect. *Proliferae* is located, revealing that sect. *Proliferae*, *Crystallophlomis*, and *Petiolares* are not monophyletic groups, which is inconsistent with traditional taxonomy (Hu, 1990). 13 species of the sect. *Proliferae* are clustered into two branches, one including *P. mallophylla*, *P. stenodonta*, *P. bulleyana*, *P. beesiana*, and *P. chungensis*, and the rest are in the other. The sect. *Amethyatina* and *Petiolares* nested in the *Proliferae* have been agreed upon in studies using the chloroplast genome (Xie et al., 2023). In morphology, the three sections also have similar characteristics, such as umbel, spherical capsule, bell calyx (Hu, 1990). However, in the nuclear gene phylogenetic trees or joint phylogenetic trees of nuclear and chloroplast genes, the species of the sect. *Proliferae* cluster into a monophyletic lineage (Liu, 2022; Yan et al., 2015), reflecting the conflict of phylogenetic signaling of nucleoplasmic genes. This phenomenon was also found in a comparison of whole genomes and chloroplast genomes of 80 individuals of Asian butternuts (Xu et al., 2021). The isolation-by-distance analysis and gene flow estimation indicated that, relative to seeds, pollen exhibited significantly higher gene flow levels. This may have impeded lineage differentiation and facilitated greater interaction of the nuclear genome compared to the chloroplast genome, resulting in discordant nucleoplasmic gene phylogenetic signals. With the interspecific hybridization, parallel evolution, gene introgression, and other complex phenomena occurring frequently, the phylogenetic relationship of *Primula* is becoming complex. Therefore, conducting genome-wide phylogenetic analyses of species is essential for acquiring a more thorough comprehension of their evolutionary history and elucidating the fundamental biological mechanisms by incorporating genomic variations.

## 5 Conclusions

In this study, we report high-quality mitochondrial genome and chloroplast genome structural features of *P. mallophylla* for the first time, including repeat sequences, RNA editing events, codon preference, and intracellular gene transfer. Combined analyses of *Ka/Ks* ratios and branch-site tests reveal the evolutionary rates and adaptive evolution features of *P. mallophylla* at the organelle genome level. The phylogenetic results showed that the *P. stenodonta* is sister to the *P. mallophylla*, and the sect. *Proliferae* in which it is found is not a monophyletic group. The collinear analysis with the relatives indicated that the mitochondrial genome of *P. mallophylla* experienced genomic rearrangement. However, the chloroplast genome is less different from its relatives in sequence comparison and boundary analysis, and the overall structure is more conservative. The results of this study not only provide molecular evidence for clarifying the taxonomic status and reconstructing the evolutionary background of *P. mallophylla*, but also provide important references for the conservation of germplasm resources and systematic evolution of endangered plants.

## Data Availability

The mitochondrial and chloroplast genome sequences of *P. mallophylla* generated in this study have been deposited in the NCBI GenBank database (https://www.ncbi.nlm.nih.gov/) under the accession numbers PV527762.1 and PQ31024.1, respectively. All raw data have been stored in the SRA database (BioProject: PRJNA1260928; BioSample: SAMN48407255; SRA: SRR33482105, SRR33482106).
