# Supplementary material for: Organelle genomes reveal adaptive evolution and phylogenetic position of the endangered Primula mallophylla
Source: Front Plant Sci. 2025 Oct 28;16:1653128. doi: 10.3389/fpls.2025.1653128 (PMC12602451; doi:10.3389/fpls.2025.1653128)
Supplement: Supplementary file 1 [file DataSheet1.docx]

Supplementary Material

# Supplementary Figures and Tables

## Supplementary Figures


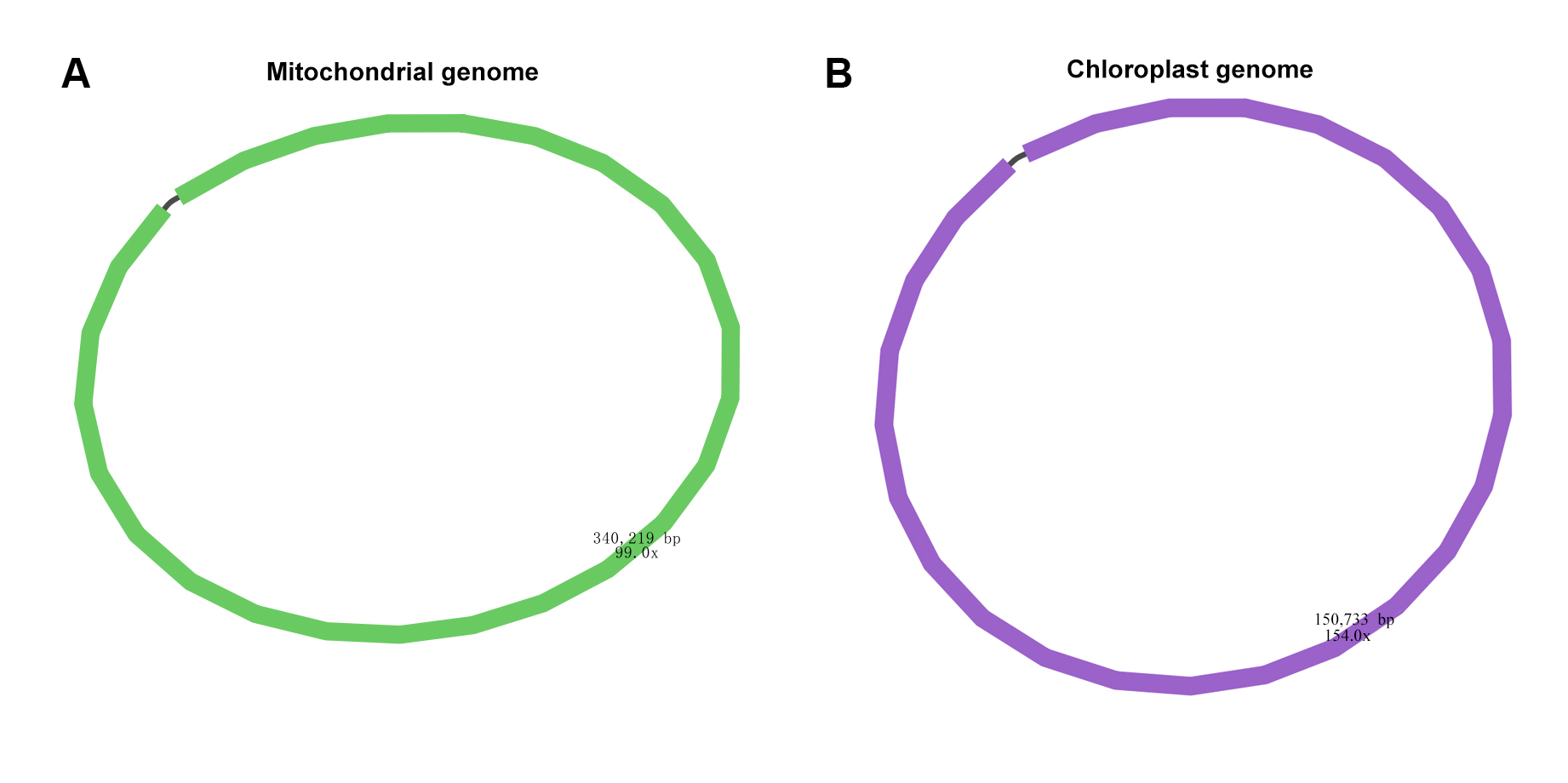


**Supplementary Figure S1.** Schematic diagram of the mitochondrial **(A)** and chloroplast **(B)** genomes assemblies of *P. mallophylla*.


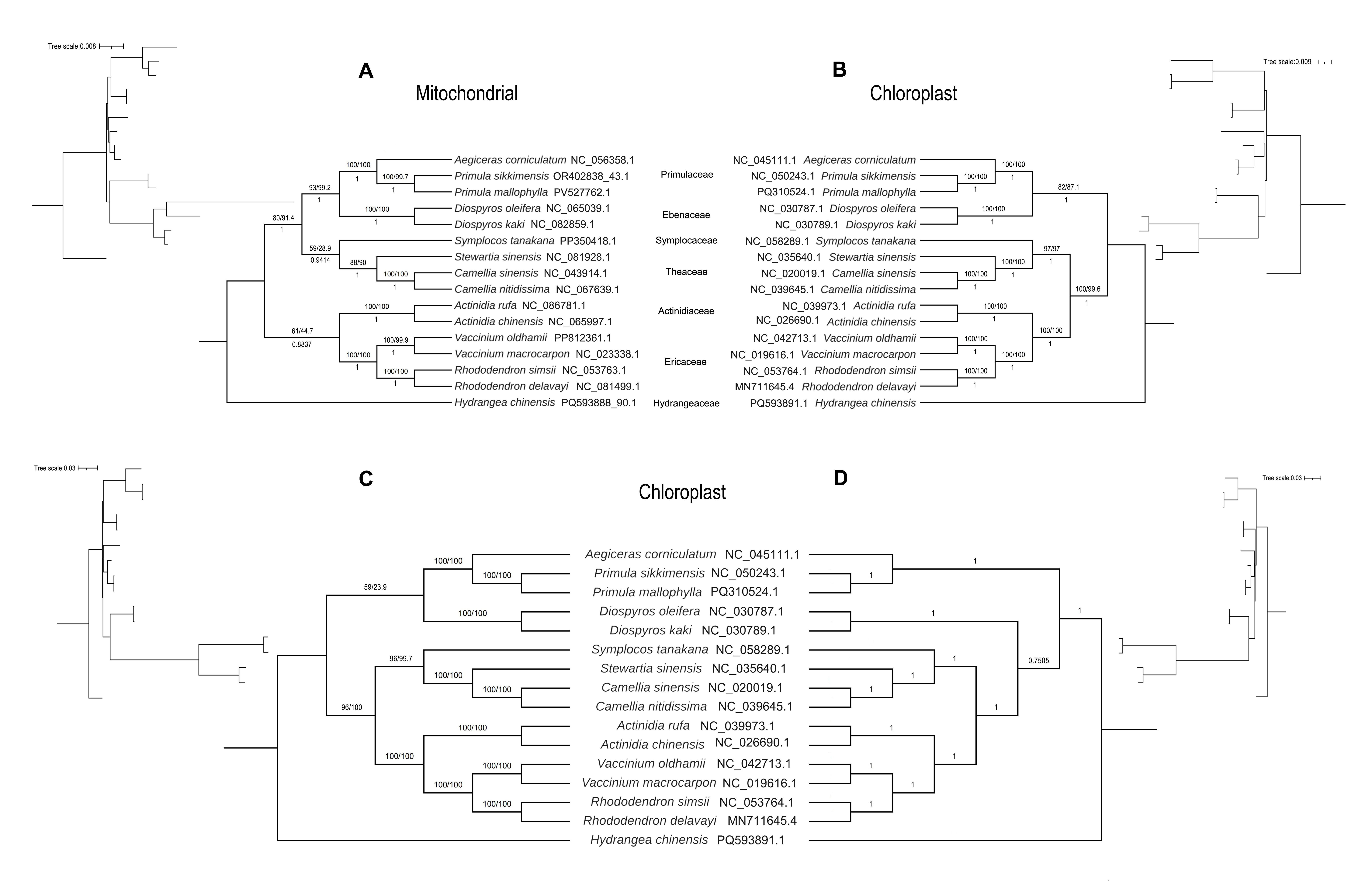


**Supplementary Figure S2.** Phylogenetic analyses of 16 species based on mitochondrial and chloroplast genomes using different tree-building methods and strategies. Codon-partitioned phylogenetic trees (ML and BI) constructed from mitochondrial (**A**) and chloroplast (**B**) PCGs of 16 species, and phylogenetic trees based on complete chloroplast genomes, including ML (**C**) and BI (**D**) trees of the same 16 species. Branches show bootstrap/SH-aLRT support values above and Bayesian posterior probabilities below. NCBI accession numbers for each species are indicated.





**Supplementary Figure S3.** Selection pressure analysis of mitochondrial and chloroplast PCGs in 16 species. (**A-C**) Density distributions of *Ka*, *Ks*, and *Ka/Ks* ratios for PCGs in mitochondrial (Mt) and chloroplast (Cp) genomes. (**D**) and (**E**) show boxplots of *Ka/Ks* values for mitochondrial and chloroplast PCGs, respectively.





**Supplementary Figure S4.** **Selection pressure analysis of chloroplast PCGs in 15 Primulaceae species.** (**A-C**) Density distributions of *Ka,* *Ks*, and *Ka/Ks* ratios for chloroplast PCGs. (**D**) Boxplot of *Ka/Ks* values for PCGs.


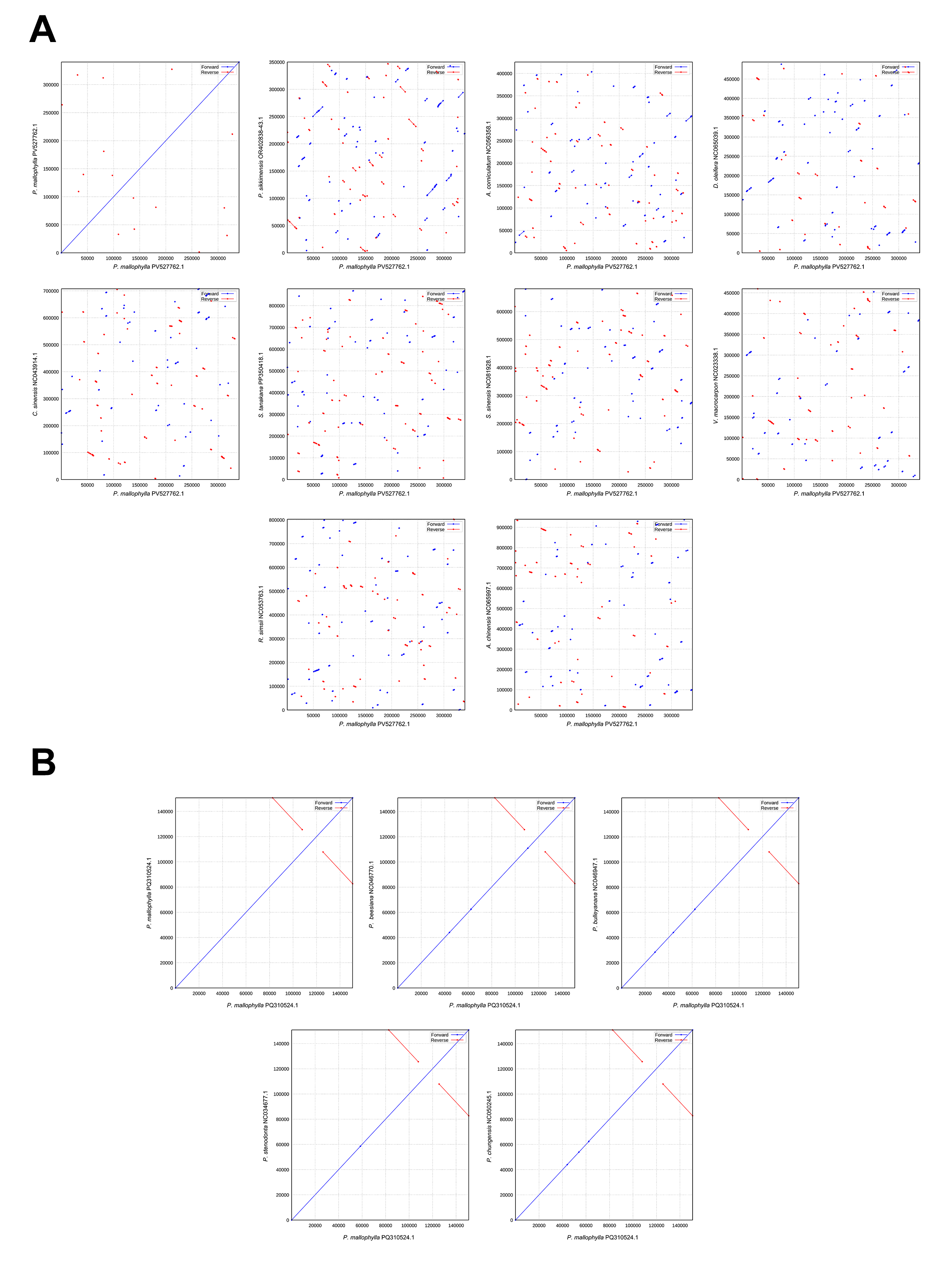


**Supplementary Figure S5.** Dotplots of *P. mallophylla* and its closely related species. (**A**) Mitochondrial genomes dotplots. (**B**) Chloroplast genomes dotplots. In both plots, blue lines indicate regions with the same orientation, and red lines indicate inverted regions.

## Supplementary Tables

**Supplementary Table S1.** Information of each species in phylogenetic trees of Ericales.

| **Species** | **Order** | **Family** | **Accession number of mitochondrial genomes** | **Accession number of chloroplast genomes** |
| --- | --- | --- | --- | --- |
| *Primula mallophylla* | Ericales | Primulaceae | PV527762.1 | PQ310524.1 |
| *Primula sikkimensis* | Ericales | Primulaceae | NC_082937.1-43.1 | NC_050243.1 |
| *Aegiceras corniculatum* | Ericales | Primulaceae | NC_056358.1 | NC_045111.1 |
| *Stewartia sinensis* | Ericales | Theaceae | NC_081928.1 | NC_035640.1 |
| *Camellia nitidissima* | Ericales | Theaceae | NC_067639.1 | NC_039645.1 |
| *Camellia sinensis* | Ericales | Theaceae | NC_043914.1 | NC_020019.1 |
| *Symplocos tanakana* | Ericales | Symplocaceae | PP350418.1 | NC_058289.1 |
| *Actinidia chinensis* | Ericales | Actinidiaceae | NC_065997.1 | NC_026690.1 |
| *Actinidia rufa* | Ericales | Actinidiaceae | NC_086781.1 | NC_039973.1 |
| *Diospyros oleifera* | Ericales | Ebenaceae | NC_065039.1 | NC_030787.1 |
| *Diospyros kaki* | Ericales | Ebenaceae | NC_082859.1 | NC_030789.1 |
| *Rhododendron simsii* | Ericales | Ericaceae | NC_053763.1 | NC_053764.1 |
| *Rhododendron delavayi* | Ericales | Ericaceae | NC_081499.1 | MN711645.4 |
| *Vaccinium macrocarpon* | Ericales | Ericaceae | NC_023338.1 | NC_019616.1 |
| *Vaccinium oldhamii* | Ericales | Ericaceae | PP812361.1 | NC_042713.1 |
| *Hydrangea chinensis* | Cornales | Hydrangeaceae | PQ593888.1-90.1 | PQ593891.1 |

**Supplementary Table S2.** Information of each species in the phylogenetic trees of *Primula*.

| **Species** | **Genus** | **Section** | **Accession number of chloroplast genomes** |
| --- | --- | --- | --- |
| *Primula cavaleriei* | *Primula* | *Monocarpicae* | NC_088048.1 |
| *Primula duclouxii* | *Primula* | *Monocarpicae* | NC_058263.1 |
| *Primula effusa* | *Primula* | *Monocarpicae* | NC_058259.1 |
| *Primula forbesii* | *Primula* | *Monocarpicae* | NC_061696.1 |
| *Primula pellucida* | *Primula* | *Monocarpicae* | NC_050248.1 |
| *Primula persimilis* | *Primula* | *Monocarpicae* | NC_034331.1 |
| *Primula tsiangii* | *Primula* | *Monocarpicae* | NC_046755.1 |
| *Primula filchnerae* | *Primula* | *Auganthus* | NC_051972.1 |
| *Primula sinensis* | *Primula* | *Auganthus* | NC_030609.1 |
| *Primula cawdoriana* | *Primula* | *Soldanelloides* | NC_053586.1 |
| *Primula flaccida* | *Primula* | *Soldanelloides* | NC_053595.1 |
| *Primula hookeri* | *Primula* | *Petiolares* | NC_053593.1 |
| *Primula moupinensis* | *Primula* | *Petiolares* | NC_050244.1 |
| *Primula odontocalyx* | *Primula* | *Petiolares* | NC_065386.1 |
| *Primula ovalifolia* | *Primula* | *Petiolares* | NC_064961.1 |
| *Primula sinuata* | *Primula* | *Petiolares* | NC_088046.1 |
| *Primula sonchifolia* | *Primula* | *Petiolares* | NC_053594.1 |
| *Primula strumosa* | *Primula* | *Petiolares* | NC_053599.1 |
| *Primula taliensis* | *Primula* | *Petiolares* | NC_053601.1 |
| *Primula chapaensis* | *Primula* | *Carolinella* | NC_088043.1 |
| *Primula kwangtungensis* | *Primula* | *Carolinella* | NC_034371.1 |
| *Primula kweichouensis* | *Primula* | *Carolinella* | NC_088047.1 |
| *Primula zhui* | *Primula* | *Carolinella* | NC_088044.1 |
| *Primula anisodora* | *Primula* | *Proliferae* | NC_053578.1 |
| *Primula beesiana* | *Primula* | *Proliferae* | NC_046770.1 |
| *Primula bulleyana* | *Primula* | *Proliferae* | NC_046947.1 |
| *Primula chrysochlora* | *Primula* | *Proliferae* | NC_034678.1 |
| *Primula chungensis* | *Primula* | *Proliferae* | NC_050245.1 |
| *Primula helodoxa* | *Primula* | *Proliferae* | NC_046771.1 |
| *Primula miyabeana* | *Primula* | *Proliferae* | NC_053607.1 |
| *Primula poissonii* | *Primula* | *Proliferae* | NC_024543.1 |
| *Primula secundiflora* | *Primula* | *Proliferae* | NC_053585.1 |
| *Primula smithiana* | *Primula* | *Proliferae* | NC_061709.1 |
| *Primula stenodonta* | *Primula* | *Proliferae* | NC_034677.1 |
| *Primula wilsonii* | *Primula* | *Proliferae* | MW442886.1 |
| *Primula ambita* | *Primula* | *Obconicolisteri* | NC_058260.1 |
| *Primula asarifolia* | *Primula* | *Obconicolisteri* | NC_058256.1 |
| *Primula densa* | *Primula* | *Obconicolisteri* | NC_058262.1 |
| *Primula dumicola* | *Primula* | *Obconicolisteri* | NC_058257.1 |
| *Primula obconica* | *Primula* | *Obconicolisteri* | NC_046415.1 |
| *Primula oreodoxa* | *Primula* | *Obconicolisteri* | NC_050848.1 |
| *Primula rubifolia* | *Primula* | *Obconicolisteri* | NC_058261.1 |
| *Primula vilmoriniana* | *Primula* | *Obconicolisteri* | NC_058258.1 |
| *Primula algida* | *Primula* | *Aleuritia* | NC_053582.1 |
| *Primula gemmifera* | *Primula* | *Aleuritia* | NC_053590.1 |
| *Primula knuthiana* | *Primula* | *Aleuritia* | NC_039350.1 |
| *Primula pulchella* | *Primula* | *Aleuritia* | NC_050246.1 |
| *Primula stenocalyx* | *Primula* | *Aleuritia* | NC_058249.1 |
| *Primula annulata* | *Primula* | *Minutissimae* | NC_053608.1 |
| *Primula walshii* | *Primula* | *Minutissimae* | NC_053597.1 |
| *Primula cicutariifolia* | *Primula* | *Ranunculoides* | NC_053605.1 |
| *Primula jiugongshanensis* | *Primula* | *Ranunculoides* | NC_056335.1 |
| *Primula merrilliana* | *Primula* | *Ranunculoides* | NC_053604.1 |
| *Primula qiupuensis* | *Primula* | *Ranunculoides* | NC_064959.1 |
| *Primula ranunculoides* | *Primula* | *Ranunculoides* | NC_056361.1 |
| *Primula wannanensis* | *Primula* | *Ranunculoides* | NC_064960.1 |
| *Primula veris* | *Primula* | *Primtula* | NC_031428.1 |
| *Primula erythrocarpa* | *Primula* | *Denticulata* | NC_053598.1 |
| *Primula homogama* | *Primula* | *Souliei* | NC_054305.1 |
| *Primula vialii* | *Primula* | *Muscarioides* | NC_065387.1 |
| *Primula capitata* | *Primula* | *Capitatae Pax* | NC_053589.1 |
| *Primula hubeiensis* | *Primula* | *Auganthus* | NC_056372.1 |
| *Primula calliantha* | *Primula* | *Crystallophlomis* | MZ054238.1 |
| *Primula chionantha* | *Primula* | *Crystallophlomis* | NC_053583.1 |
| *Primula handeliana* | *Primula* | *Crystallophlomis* | NC_039348.1 |
| *Primula purdomii* | *Primula* | *Crystallophlomis* | NC_053591.1 |
| *Primula szechuanica* | *Primula* | *Crystallophlomis* | NC_080275.1 |
| *Primula woodwardii* | *Primula* | *Crystallophlomis* | NC_039349.1 |
| *Primula dryadifolia* | *Primula* | *Dryadifoiia* | NC_053596.1 |
| *Primula medogensis* | *Primula* | *Cordifoliae* | PP860589.1 |
| *Primula geraniifolia* | *Primula* | *Cortusoides* | NC_053600.1 |
| *Primula heucherifolia* | *Primula* | *Cortusoides* | NC_053580.1 |
| *Primula saxatilis* | *Primula* | *Cortusoides* | NC_053584.1 |
| *Primula septemloba* | *Primula* | *Cortusoides* | NC_053603.1 |
| *Primula sieboldii* | *Primula* | *Cortusoides* | NC_085672.1 |
| *Primula violaris* | *Primula* | *Cortusoides* | NC_058572.1 |
| *Primula alpicola* | *Primula* | *Sikkimensis* | NC_053588.1 |
| *Primula florindae* | *Primula* | *Sikkimensis* | NC_053579.1 |
| *Primula sikkimensis* | *Primula* | *Sikkimensis* | NC_050243.1 |
| *Primula waltonii* | *Primula* | *Sikkimensis* | NC_058808.1 |
| *Primula bracteata* | *Primula* | *Bullatae* | NC_053592.1 |
| *Primula forrestii* | *Primula* | *Bullatae* | NC_053602.1 |
| *Primula amethystina* | *Primula* | *Amethyatina* | NC_053577.1 |
| *Primula faberi* | *Primula* | *Amethyatina* | NC_053576.1 |
| *Primula virginis* | *Primula* | *Amethyatina* | NC_053581.1 |
| *Primula mallophylla* | *Primula* | *Proliferae Pax* | PQ310524.1 |
| *Androsace filiformis* | *Androsace* | *Androsace* | NC_080502.1 |
| *Androsace axillaris* | *Androsace* | *Samuelia* | NC_080505.1 |

**Supplementary Table S3.** Information on phylogenetic tree construction.

| **Dataset** | **PCGs** | **Strategy** | **Method** | **Best-fit nucleotide substitution model** |
| --- | --- | --- | --- | --- |
| Chloroplast PCGs of 16 species | *atpA, atpB, atpE, atpH, atpI, cemA, ndhA, ndhB, ndhC, ndhD, ndhE, ndhF, ndhH, ndhI, ndhJ, petA, petB, petD, petG, petL, petN, psaA, psaB, psaC, psaI, psbB, psbC, psbD, psbE, psbF, psbH, psbI, psbJ, psbK, psbL, psbM, psbN, psbT, rbcL, rpl14, rpl22, rpl23, rpl2, rpl32, rpl33, rpl36, rpoA, rpoB, rpoC1, rpoC2, rps11, rps14, rps15, rps18, rps2, rps3, rps4, rps7, rps8* | DNA | ML | TVM+F+I+R2 |
|  |  | DNA | BI | GTR+F+I+G4 |
|  |  | codon | ML | TVM+F+I+R2 |
|  |  | codon | BI | GTR+F+I+G4 |
| Mitochondrial PCGs of 16 species | *atp1, atp4, atp9, ccmB, ccmC, ccmFC, ccmFN, cob, cox2, matR, nad3, nad4, nad5, nad6, rps12* | DNA | ML | GTR+F+G4 |
|  |  | DNA | BI | GTR+F+I+G4 |
|  |  | codon | ML | GTR+F+G4 |
|  |  | codon | BI | GTR+F+I+G4 |
| Chloroplast genomes of 88 species | - | DNA | ML | TVM+F+I+R7 |
|  |  | DNA | BI | GTR+F+I+G1 |
| Chloroplast PCGs of 88 species | *atpA, atpB, atpE, atpF, atpH, atpI, ccsA, clpP, matK, ndhA, ndhB, ndhC, ndhE, ndhF, ndhG, ndhH, ndhI, ndhJ, ndhK, petA, petB, petD, petG, petL, petN, psaA, psaB, psaC, psaI, psaJ, psbA, psbB, psbC, psbD, psbE, psbF, psbH, psbJ, psbK, psbL, psbM, psbT, psbZ, rbcL, rpl14, rpl20, rpl22, rpl23, rpl32, rpl33, rpl36, rpoA, rpoB, rpocL, rps11, rps12, rps14, rps15, rps16, rps2, rps3, rps4, rps7, rps8, ycf2* | DNA | ML | TVM+F+I+R3 |
|  |  | DNA | BI | GTR+F+I+G1 |
| Chloroplast PCGs of 15 species  (*P. mallophylla, P. forbesii, P. filchnerae, P. odontocalyx, P. beesiana, P. chungensis, P. poissonii, P. stenodonta, P. obconica, P. pulchella, P. veris, P. handeliana, P. sikkimensis, Aegiceras corniculatum, Diospyros oleifera*) | *atpA, atpB, atpE, atpF, atpH, atpI, ccsA, cemA, clpP, matK, ndhA, ndhB, ndhC, ndhD, ndhE, ndhF, ndhG, ndhH, ndhI, ndhJ, ndhK, petA, petB, petD, petG, petL, petN, psaA, psaB, psaC, psaI, psaJ, psbA, psbB, psbC, psbD, psbE, psbF, psbH, psbI, psbJ, psbK, psbL, psbM, psbN, psbT, psbZ, rbcL, rpl14, rpl16, rpl2, rpl20, rpl22, rpl23, rpl32, rpl33, rpl36, rpoA, rpoB, rpoC1, rpoC2, rps11, rps12, rps14, rps15, rps16, rps18, rps19, rps2, rps3, rps4, rps7, rps8, ycf1, ycf2* | DNA | ML | TVM+F+R3 |

**Supplementary Table S4.** Gene composition in the mitochondrial and chloroplast genome of *P. mallophylla*.

| Types of organelles | Group of genes | Name of genes |
| --- | --- | --- |
| Mitochondrion | ATP synthase | *atp1, atp4, atp6, atp8, atp9* |
|  | NADH dehydrogenase | *nad1, nad2, nad3, nad4, nad4L, nad5, nad6, nad7, nad9* |
|  | Cytochrome *b* | *cob* |
|  | Cytochrome *c* biogenesis | *ccmB, ccmC, ccmFC, ccmFN* |
|  | Cytochrome *c* oxidase | *cox1, cox2, cox3* |
|  | Maturases | *matR* |
|  | Protein transport subunit | *mttB* |
|  | Ribosomal protein large subunit | *rpl2, rpl5, rpl10, rpl16* |
|  | Ribosomal protein small subunit | *rps1, rps3, rps4, rps10, rps12, rps13, rps14* |
|  | Succinate dehydrogenase | *sdh3, sdh4* |
|  | Ribosome RNA | *rrn5, rrn18, rrn26* |
|  | Transfer RNA | *trnA-UGC, trnC-GCA, trnD-GUC, trnE-UUC, trnF-GAA, trnfM-CAU(×2), trnG-GCC, trnH-GUG, trnI-CAU, trnK-UUU, trnL-CAA, trnM-CAU, trnN-GUU, trnP-UGG, trnQ-UUG, trnS-CGA, trnS-GCU, trnS-UGA, trnV-GAC, trnW-CCA, trnY-GUA* |
| Chloroplast | ATP synthase subunit | *atpA, atpB, atpE, atpF, atpH, atpI* |
|  | Photosystem I subunit | *psaA, psaB, psaC, psaI, psaJ* |
|  | Photosystem II subunit | *psbA, psbB, psbC, psbD, psbE, psbF, psbI, psbJ, psbK, psbL, psbM, psbN, psbT, psbZ, ycf3* |
|  | NADH-dehydrogenase subunit | *ndhA, ndhB(×2), ndhC, ndhD, ndhE, ndhF, ndhG, ndhH, ndhI, ndhJ, ndhK* |
|  | Cytochrome b/f complex subunit | *petA, petB, petD, petG, petL, petN* |
|  | Rubisco | *rbcL* |
|  | Ribosomal RNA | *rrn4.5(×2), rrn5 (×2), rrn16(×2), rrn23(×2)* |
|  | Transfer RNA | *trnA-UGC(×2), trnC-GCA, trnD-GUC, trnE-UUC, trnF-GAA, trnfM-CAU, trnG-UCC(×2), trnH-GUG, trnI-CAU(×2), trnI-GAU(×2), trnK-UUU, trnL-CAA(×2), trnL-UAA, trnL-UAG, trnM-CAU, trnN-GUU(×2), trnP-UGG, trnQ-UUG, trnR-ACG(×2), trnR-UCU, trnS-GCU, trnS-GGA, trnS-UGA, trnT-GGU, trnT-UGU, trnV-GAC(×2), trnV-UAC, trnW-CCA, trnY-GUA* |
|  | Ribosomal protein large subunit | *rpl2(×2), rpl14, rpl16, rpl20, rpl22, rpl23(×2), rpl32, rpl33, rpl36* |
|  | Ribosomal protein small subunit | *rps2, rps3, rps4, rps7(×2), rps8, rps11, rps12(×2), rps14, rps15, rps16, rps18, rps19* |
|  | DNA dependent RNA polymerase | *rpoA, rpoB, rpoC1, rpoC2* |
|  | Other genes | *accD, ccsA, cemA, clpP, matK* |
|  | Genes of unknown function | *ycf1, ycf2(×2), ycf4, ycf15(×2)* |

**Supplementary Table S5.** RNA editing site prediction in the mitochondrial genome of *P. mallophylla*.

| Type | Amino change | Number | Percentage |
| --- | --- | --- | --- |
| Hydrophilic-hydrophilic | Asn-Asn | 1 | 14.11% |
|  | His-Tyr | 26 |  |
|  | Tyr-Tyr | 1 |  |
|  | Arg-Cys | 35 |  |
|  | Ser-Ser | 3 |  |
|  | Thr-Thr | 1 |  |
| Hydrophilic-hydrophobic | Arg-Trp | 30 | 44.21% |
|  | Thr-Ile | 8 |  |
|  | Ser-Leu | 104 |  |
|  | Ser-Phe | 68 |  |
| Hydrophilic-stop | Gln-End | 1 | 1.47% |
|  | Arg-End | 1 |  |
|  | Thr-Met | 5 |  |
| Hydrophobic-hydrophilic | Pro-Ser | 36 | 7.58% |
| Hydrophobic-hydrophobic | Ala-Val | 5 | 32.63% |
|  | Ile-Ile | 2 |  |
|  | Leu-Leu | 4 |  |
|  | Leu-Phe | 17 |  |
|  | Pro-Leu | 105 |  |
|  | Pro-Phe | 14 |  |
|  | Pro-Pro | 2 |  |
|  | Val-Val | 1 |  |
|  | Phe-Phe | 5 |  |

**Supplementary Table S6.** RSCU of codons corresponding to amino acids in the organelle genomes of *P. mallophylla*.

| Amino  acid | RSCU of the mitochondrial genome | | | | | | RSCU of the chloroplast genome | | | | | |
| --- | --- | --- | --- | --- | --- | --- | --- | --- | --- | --- | --- | --- |
| Ala | GCU | GCA | GCC | GCG |  |  | GCU | GCA | GCC | GCG |  |  |
|  | 1.6 | 1.02 | 0.91 | 0.47 |  |  | 1.85 | 1.16 | 0.57 | 0.42 |  |  |
| Arg | AGA | CGA | CGU | AGG | CGG | CGC | AGA | CGA | CGU | AGG | CGG | CGC |
|  | 1.46 | 1.38 | 1.2 | 0.72 | 0.66 | 0.57 | 1.84 | 1.44 | 1.31 | 0.54 | 0.49 | 0.37 |
| Asn | AAU | AAC |  |  |  |  | AAU | AAC |  |  |  |  |
|  | 1.34 | 0.66 |  |  |  |  | 1.58 | 0.42 |  |  |  |  |
| Asp | GAU | GAC |  |  |  |  | GAU | GAC |  |  |  |  |
|  | 1.4 | 0.6 |  |  |  |  | 1.61 | 0.39 |  |  |  |  |
| Cys | UGU | UGC |  |  |  |  | UGU | UGC |  |  |  |  |
|  | 1.23 | 0.77 |  |  |  |  | 1.51 | 0.49 |  |  |  |  |
| Gln | CAA | CAG |  |  |  |  | CAA | CAG |  |  |  |  |
|  | 1.54 | 0.46 |  |  |  |  | 1.52 | 0.48 |  |  |  |  |
| Glu | GAA | GAG |  |  |  |  | GAA | GAG |  |  |  |  |
|  | 1.37 | 0.63 |  |  |  |  | 1.53 | 0.47 |  |  |  |  |
| Gly | GGA | GGU | GGG | GGC |  |  | GGA | GGU | GGG | GGC |  |  |
|  | 1.47 | 1.33 | 0.7 | 0.5 |  |  | 1.55 | 1.4 | 0.59 | 0.47 |  |  |
| His | CAU | CAC |  |  |  |  | CAU | CAC |  |  |  |  |
|  | 1.51 | 0.49 |  |  |  |  | 1.54 | 0.46 |  |  |  |  |
| Ile | AUU | AUA | AUC |  |  |  | AUU | AUA | AUC |  |  |  |
|  | 1.3 | 0.89 | 0.81 |  |  |  | 1.53 | 0.93 | 0.54 |  |  |  |
| Leu | UUA | CUU | UUG | CUA | CUC | CUG | UUA | CUU | UUG | CUA | CUG | CUC |
|  | 1.48 | 1.3 | 1.21 | 0.96 | 0.55 | 0.5 | 2.03 | 1.27 | 1.19 | 0.79 | 0.37 | 0.35 |
| Lys | AAA | AAG |  |  |  |  | AAA | AAG |  |  |  |  |
|  | 1.19 | 0.81 |  |  |  |  | 1.54 | 0.46 |  |  |  |  |
| Met | AUG |  |  |  |  |  | AUG |  |  |  |  |  |
|  | 1 |  |  |  |  |  | 1 |  |  |  |  |  |
| Phe | UUU | UUC |  |  |  |  | UUU | UUC |  |  |  |  |
|  | 1.16 | 0.84 |  |  |  |  | 1.39 | 0.61 |  |  |  |  |
| Pro | CCU | CCA | CCC | CCG |  |  | CCU | CCA | CCC | CCG |  |  |
|  | 1.51 | 1.1 | 0.77 | 0.63 |  |  | 1.58 | 1.14 | 0.81 | 0.48 |  |  |
| Ser | UCU | UCA | AGU | UCC | UCG | AGC | UCU | AGU | UCA | UCC | UCG | AGC |
|  | 1.44 | 1.16 | 1.05 | 0.92 | 0.8 | 0.63 | 1.79 | 1.3 | 1.22 | 0.86 | 0.52 | 0.32 |
| Thr | ACU | ACC | ACA | ACG |  |  | ACU | ACA | ACC | ACG |  |  |
|  | 1.39 | 1.01 | 0.97 | 0.63 |  |  | 1.73 | 1.19 | 0.64 | 0.43 |  |  |
| Trp | UGG |  |  |  |  |  | UGG |  |  |  |  |  |
|  | 1 |  |  |  |  |  | 1 |  |  |  |  |  |
| Tyr | UAU | UAC |  |  |  |  | UAU | UAC |  |  |  |  |
|  | 1.53 | 0.47 |  |  |  |  | 1.66 | 0.34 |  |  |  |  |
| Val | GUA | GUU | GUG | GUC |  |  | GUA | GUU | GUG | GUC |  |  |
|  | 1.26 | 1.21 | 0.86 | 0.68 |  |  | 1.55 | 1.5 | 0.5 | 0.45 |  |  |
| End | UAA | UGA | UAG |  |  |  | UAA | UGA | UAG |  |  |  |
|  | 1.37 | 1.2 | 0.43 |  |  |  | 1.82 | 0.71 | 0.47 |  |  |  |

**Supplementary Table S7.** Analysis of homologous fragments based on the *P. mallophylla*.

| **Number** | **Identity (%)** | **Alignment Length (bp)** | **MTPT annotation** |
| --- | --- | --- | --- |
| MTPT1 | 95.89 | 535 | partial *trn*A-UGC; partial *trnI*-GAU |
| MTPT2 | 95.60 | 728 | complete *ycf*15; partial ycf2 |
| MTPT3 | 95.46 | 88 | complete *trn*N-GUU |
| MTPT4 | 95.24 | 84 | complete *trn*D-GUC |
| MTPT5 | 95.05 | 101 | complete *trn*W-CCA |
| MTPT6 | 91.14 | 79 | complete *trn*M-CAU |
| MTPT7 | 91.10 | 146 | partial *psb*C |
| MTPT8 | 87.30 | 189 | partial *atp*A |
| MTPT9 | 87.18 | 78 | complete *trn*I-CAU |

**Supplementary Table S8.** Genes under significant positive selection identified by branch-site analysis based on different phylogenetic tree topologies.

| **Phylogenetic tree** | **foreground branch** | **gene** | **LRT** | **pvalue** |
| --- | --- | --- | --- | --- |
| phylogenetic tree of chloroplast PCGs from 16 species | *Primula* | *rpl22* | 11.51 | 0.00069 |
|  |  | *rbcL* | 8.40 | 0.00374 |
| phylogenetic tree of chloroplast PCGs from 15 species | *Primula mallophylla* | *ndhB* | 11.11 | 0.00086 |
|  |  | *rpl2* | 7.87 | 0.00504 |
|  |  |  |  |  |
